# Attitudes of Physicians and Individuals Toward Digital Mental Health Tools: Protocol for a Web-Based Survey Research Project

**DOI:** 10.2196/41040

**Published:** 2023-03-14

**Authors:** Diogo Nogueira-Leite, Ricardo Cruz-Correia

**Affiliations:** 1 Department of Community Medicine, Information and Decision in Health Faculty of Medicine University of Porto Porto Portugal; 2 Nova School of Business and Economics Health and Economics Knowledge Center New University of Lisbon Lisbon Portugal; 3 Programme in Health Data Science Faculty of Medicine University of Porto Porto Portugal; 4 Center for Health Technology and Services Research Porto Portugal

**Keywords:** mental health, mobile apps, digital technology, government regulation

## Abstract

**Background:**

Digital transformation is impacting health care delivery. Great market dynamism is bringing opportunities and concerns alike into public discussion. Digital health apps are a vibrant segment where regulation is emerging, with Germany paving the way with its DiGA (Digitale Gesundheitsanwendungen, in German, meaning digital health apps) program. Simultaneously, mental ill-health constitutes a global health concern, and prevalence is expected to worsen due to the COVID-19 pandemic and its containment measures. Portugal and its National Health System may be a useful testbed for digital health interventions.

**Objective:**

The paper outlines the protocol for a research project on the attitudes of physicians and potential users toward digital mental health apps to improve access to care, patient outcomes, and reduce the burden of disease of mental ill-health.

**Methods:**

Web surveys will be conducted to acquire data from the main stakeholders (physicians and the academic community). Data analysis will replicate the statistical analysis performed in the studies from Dahlhausen and Borghouts to derive conclusions regarding the relative acceptance and likelihood of successful implementation of digital mental health apps in Portugal.

**Results:**

The findings of the proposed studies will elicit important information on how physicians and individuals perceive digital mental health app interventions to improve access to care, patient outcomes, and reduce the burden of disease of mental ill-health. Data collection ran between September 26 and November 6, 2022, for the first study and September 20 and October 20, 2022, for the second study. We obtained 160 responses to the first study’s survey and 539 answers to the second study’s survey. Data analysis is concluded, and both studies’ results are expected to be published in 2023.

**Conclusions:**

The results of the studies projected in this research protocol will have implications for researchers and academia, industry, and policy makers concerning the adoption and implementation of digital health mental apps and associated interventions.

**International Registered Report Identifier (IRRID):**

DERR1-10.2196/41040

## Introduction

### Digital Transformation in Health

Digital transformation and its impact on health [1] is evermore present [2]. Investment inflow into the digital transformation in the health care market registered a 10.4% compounded annual growth rate from 2016 to 2020, with a further 14% compounded annual growth rate expected in 2021-2031, denoting strong market dynamics [3]. According to IQVIA Institute for Human Data Science’s Digital Health Trends 2021 report [4], mobile apps in the business-to-consumer market registered at “(…) top app stores worldwide are now over 350,000 in number, with more than 90,000 digital health apps added in 2020—an average of more than 250 apps per day.”

The growth of digital tools applied to health has, however, been largely achieved without regulatory scrutiny appropriate to the needs of a digital society and due consideration for the quality, safety, and effectiveness of tools. There is rising concern that mobile apps, for one, do not meet enough clinical or technical validation standards or even register any type of empirical support [5-8]. Further, following a rush of optimism as the COVID-19 pandemic became a global challenge and research organizations tried to leverage technological tools, the evidence showed that, for instance, artificial intelligence–based mechanisms often failed to deliver on their promise [9], thus making the discussion more relevant. If COVID-19 brought concerns about the tools used for digital health delivery, it is also true that it provided a very significant impulse to their adoption, laying the ground for their widespread use in a postpandemic world [10], including in fields such as digital mental health (MH) [11].

Physicians and prospective patients alike have several concerns regarding digital health, and there are myriad challenges and risks ahead. These include lack of digital literacy, barriers to health care access that stem from the digital divide, and worries about losing touch with a familiar model such as face-to-face consultation [12-16]. Further questions arise around legal and ethical questions around topics such as confidentiality, privacy, patient autonomy, or the trustworthiness and accountability of care delivery [17-20].

The scope of our research protocol encompasses 2 different concepts that need clarification: digital health and, more particularly, digital mental health apps (DMHAs). This clarification is important as none of these constructs has a stabilized definition due both to the evolving nature of their underlying fields and to the heterogeneity of stakeholders that work with them, ranging from academia to governmental institutions and industry.

For this research protocol and its papers, digital health will be defined as technologies that connect and empower people and populations to manage their health and wellness by augmenting workforce capacity while leveraging digital tools and technologies to shape care delivery [21]. It uses information and communication technologies to facilitate the understanding of health problems and challenges faced by people receiving medical treatment and social prescribing in more personalized and precise ways [22-25].

Within digital health, we will be focusing on DMHAs—a type of digital MH [26] tool that uses the format of a mobile app to aid MH assessment, support, prevention, and treatment [27,28].

### The Rise of Digital Health Apps—Regulatory Concerns

Digital health apps promise to empower users and provide a unique opportunity for access to health (eg, by expanding remote care) while increasing the volume and variety of information available to physicians. More than 350,000 health apps are available from various app stores, with 110 downloaded over 10 million times and accounting for almost 50% of all downloads [4]. According to the same source, COVID-19 increased app use, and MH, cardiovascular, and diabetes condition management apps accounted for nearly 50% of disease-focused apps. However, considering the difficulty in assessing the impact of digital health apps [29] on health, policy makers have recently taken measures to reduce the uncertainty surrounding digital health apps by introducing standardized processes for evaluation akin to medicines or medical devices [30].

The identification of digital health apps that are safe and effective presents a significant challenge for health systems across the globe, making proper regulation essential to minimize risks and magnify benefits. Apps that tackle high-risk or chronic conditions, or that attempt to perform diagnosis or treatment, are especially relevant in this scenario given the time one is expected to use them or the sensitivity of associated information. Regulation constitutes an aprioristic need to produce payment and reimbursement pathways that balance costs and benefits while ensuring access and defining clear use policies aside from those that are market based (eg, star ratings in app stores) [31].

When it comes to implementing a regulatory framework aimed at digital health apps and their market access and reimbursement, Germany assumed a pioneering role. Its DiGA (Digitale Gesundheitsanwendungen, in German, meaning digital health apps) [32] program was inaugurated in October 2020, and, on December 20, 2022, 34 apps had qualified for statutory insurance reimbursement (of these, 14, or 41%, were classified as being DiGA for mental disorders) [33]. An app is accepted into the DiGA program after the German Federal Agency for Medicines and Health Products evaluates it in terms of general requisites (concerning technical safety and user-friendliness) and positive clinical effects. If an app has not yet proven the latter, it has 12 months to do so and is included in a provisory listing; if it fails to comply, it is withdrawn. If the app can prove both compliances with general requisites and positive clinical effects, German Federal Agency for Medicines and Health Products approves it, and price and reimbursement negotiations follow with the association of statutory insurers (GKV-SV), after which it is included in the DiGA Directory [33]. France is studying a replication of the DiGA approach and is expected to implement it until the end of 2022 [34].

Belgium ranks second in implementation—even though the mHealthBelgium [35] initiative was launched in 2018, it officially started conducting appraisal and reimbursement processes in January 2021. Its selection process is based on a 3-level validation pyramid [36] consisting of criteria related to legal and regulatory issues (level 1), safe communication and privacy (level 2), and financing and reimbursement (level 3, subdivided into 3^−^, if it has provisional reimbursement while proving socioeconomic benefits, and 3^+^, if it already has proven those same benefits). On June 2, 2022, 23 apps had level 1 clearance, and 12 apps had level 2 clearance. Moreover, 1 app received 3^−^ clearance, and none had been cleared for level 3^+^.

Other European countries have so far opted for softer, more decentralized approaches, with legal obligations and compliance rules based on the General Data Protection Regulation [37] or the Medical Devices Regulation [38]. Singapore and the United States, on the other hand, resort to their own medical devices regulations, with the Food and Drug Administration issuing guidance in 2021 [39] regarding a pilot project at the federal level and, thus far, assessing software as a medical device [40].

### Mental Health, Anxiety and Depression, and Pandemic: A Troublesome Cocktail

Mental disorders are globally responsible for 4.92% of total disability-adjusted life years (DALYs) and 13.04% of all disease prevalence cases. Depressive disorders are accountable for 1.84% of global DALYs and 3.76% of total disease prevalence, while anxiety answers for 1.13% of global DALYs and 4.05% of disease prevalence in the globe [41]. The 2 conditions accounted for more than half of the impact MH disorders had on global health in 2019 according to the GBD Compare tool developed by the Institute for Health Metrics and Evaluation.

Table 1 presents a breakdown of the impact mental disorders as a category, alongside anxiety and depression, had not only in global health but also on the health status of some geographies of concern for us in this protocol—the United States, the European Union, and Germany—in 2019, measured in DALY and disease prevalence cases. Data were gathered using the previously mentioned GBD Compare tool [41].

**Table 1:** Share of DALY^a^ and disease prevalence (in percentage points) per condition and geography.^b^

	World		United States		European Union		Germany	
	DALY (%)	Prevalence (%)	DALY (%)	Prevalence (%)	DALY (%)	Prevalence (%)	DALY (%)	Prevalence (%)
Mental disorders	4.92	13.04	6.56	17.03	6.65	15.34	6.43	15.59
Depression	1.84	3.76	2.38	4.92	2.42	4.6	2.16	4.32
Anxiety	1.13	4.05	1.68	6.51	1.69	5.82	1.95	7.07

^a^DALY: disability-adjusted life year.

^b^Institute for Health Metrics and Evaluation [41].

In all geographies considered in the paradigm, depression and anxiety account for over half of the burden of disease generated by mental disorders. Furthermore, these conditions are always a relevant public health issue in any of the mentioned contexts due to their prevalence, thus making the importance of addressing them undisputable.

These data are with regard to 2019, and consequently do not factor in the consequences of the COVID-19 pandemic and its containment measures in MH deterioration. Many experts have alerted to the deleterious impact [42-44] of this pandemic, especially for children and adolescents [45], and have issued calls to action on the need to tackle this issue heads on [46,47]. It has been suggested that digital MH tools could help manage the burden of the disease brought about by mental disorders, in particular anxiety and depression [48]. Rapid and affordable mechanisms of population screening and follow-up are necessary in a postpandemic world; otherwise, the burden of the disease will be unbearable for any health system [49-51].

### Portugal and Its Mental Health Landscape

The Portuguese National Health Service (NHS) is a publicly owned, tax-financed health system that aims at ensuring wide, universal, and mostly free-of-charge care [52]. Regarding total health care activity in Portugal in 2019 (the most recent and pandemic-free full-year available), the NHS delivered 59.8% of 21.1 million outpatient consultations, 76.5% of 8.2 million emergency room episodes, and performed 66.2% of 1.21 million surgeries. The NHS was also responsible for 82.1% of 180.3 million uses of complementary means of medical diagnosis and therapy, as well as for 71.5% of 1.1 million hospitalizations and 84.7% of 86.4 thousand births [53]. The Portuguese NHS was therefore accountable for 79.5% of the health care delivered in Portugal. Regarding funding, the government financed 63.8% of total health care expenditure, with out-of-pocket family spending representing 30.5% and private insurance companies accounting for 3.8% of the total [54].

The data point to an eminently public health system both in funding and activity, with significant household financial strain and a modest contribution of private insurers. It also means that most of the Portuguese population relies on the care provided by the publicly financed and managed NHS. This is a significant difference from health systems such as those of Germany [55] or the United States [56], where health and care are largely privately owned, financed, and managed. In these countries, the government only intervenes in specific cases, mostly related to income deprivation or market failures. Such differences entail different economic, managerial, and organizational incentives, reflecting different cultures and preferences [57].

Several studies draw attention to a lack of MH and care resources within the scope of the Portuguese NHS, chief among them is the first (and so far, sole) national epidemiological questionnaire on MH [58]. This constitutes an alarming background considering the Portuguese population’s particularly worrisome MH status: 51.4% of the population is expected to have a MH issue at some point in their lives. This datum reports to 2013; the Order of Portuguese Psychologists reported, in its factsheet on depression dated December 2021, that depression alone affected 10% of the population [59].

Evidence on the quasiepidemic nature of MH issues grows; the prevalence of MH disorders in 2019 was estimated at 8.27% of DALYs and 19.27% of disease cases. The statistics for anxiety and depressive disorders were, respectively, expected to be 2.58% and 3.16% of total DALYs and prevalence valued at 9.08% and 5.88% [60].

These data add to the public health relevance of these conditions, and several experts point out that, due to socioeconomic constraints ranging from stigma to lack of accessibility or affordability, these figures might be altogether underestimated [61-64]. At the same time, the World Health Organization expects the COVID-19 pandemic to have a detrimental effect on these statistics, further exacerbating the issue [65]. Children and adolescents at risk due to both family context and the COVID-19 pandemic [66-68] constitute further cause for worry.

Primary care services and digital health tools for self-management have been singled out as an opportunity to tackle this and other issues concerning health and care [69-72], namely chronic illness and quality of life, rising health costs, or significant burden of disease [73,74]. Portugal has registered a very significant uptake of telemedicine consultations [75] in its NHS, and the pandemic has arguably played a role in catalyzing this change. In context, the country ranks 16th (ie, slightly below average) in the European Commission’s 2021 Digital Economy and Society Index [76], which assesses the digital maturity of each Member State.

Unleashing the eventual potential of digital health to help manage health conditions overall and MH issues (such as anxiety and depression) in the Portuguese context implies understanding the agents, their incentives, and interconnections. The problem at its core has 2 main primary actors: physicians (defined as psychiatrists or psychologists) and patients.

Doctors are important since patients usually delegate medical decisions to doctors [77,78]; therefore, MH professionals’ attitudes toward digital health apps are a key success factor likely to steer not only adherence but also outcomes as well [79]. By the same token, we can expect doctors to be more willing to use digital tools if patients demand them. Consequently, patient buy-in is needed to make this system work, and that will only be possible if digital health developers and companies consider their expectations and concerns [80]. These can range from user interface and user experience [81] considerations to confidentiality and privacy issues [82-84].

Portugal has no direct equivalent to prescription apps or the German Ministry of Health’s DiGA program. The Portuguese reimbursement scheme for pharmaceuticals is centrally negotiated and decided by the Regulatory Authority for Medicines and Medical Devices (INFARMED). Medical devices in the European Union market are subject to a conformity assessment [38], being the definition of medical devices already extended, in law, to software and software apps. INFARMED is the authority responsible for notified bodies and has a coordinating role in verifying conformity assessments and selecting third parties to become notified bodies responsible for conformity assessments in high-risk cases.

In turn, the Shared Services of the Portuguese Ministry of Health (SPMS), largely responsible for the development of the country’s eHealth strategy, make adhesion to their health app store (MySNS Seleção [85]) conditional on an assessment system that evaluates apps concerning performance, safety, public utility, and information quality and security according to European Union Law [86]. Even though these 2 regulatory players display some level of scrutiny in European Union terms, ample room remains for economic, social, and regulatory development, showing a lag vis-à-vis German, Belgian, or French ambitions [87].

This research project is an opportunity to assess whether DMHA would be welcomed in Portugal and in a health care system with its political economy. By comparing Portugal with Germany (from the physicians’ point of view) and the United States (from the individual’s point of view) in similar settings, we hope to enrich the comparison between health systems with a very significant private sector role and systems where government plays a key function in ensuring access to and funding of health care, particularly concerning MH and the use of electronic or digital tools.

This constitutes a relevant contribution to the scientific literature not only of life sciences and their digital potential but also in terms of the industrial characterization of health economics for these subtopics and the mapping of expectations and perceptions from both supply and demand sides of MH and care.

### Aim and Objectives

The research project we refer to is composed of 2 papers, both aimed at publication in the context of a doctoral research project being pursued by the first author of this paper under the supervision of its second author.

In this research project, we aim to understand how physicians and individuals might perceive digital MH tools. These constitute, respectively, the supply and demand sides of this market. Furthermore, we seek to provide a more precise landscape of the digital health setting in Portugal and provide grounds for academic, policy, and business development. Finally, we speculate on the potential use of digital health interventions for quantifying and providing tools to tackle the increasingly unmanageable burden of mild to moderate anxiety disorder and depression.

We will try to answer the following research questions: (1) How do physicians perceive digital health apps? (2) How digitally literate do physicians perceive themselves and their patients to be? (3) How do physicians see their own role in digitalizing health care provision? (4) What are the largest benefits, risks, barriers, and opportunities in digital health apps? (5) How do individuals perceive MH apps? Which factors determine the decision of using MH apps, and how do individuals feel about doing it? (6) What are individuals’ preferences concerning resources and strategies for getting MH care? (7) How do individuals rate their MH and what impacts it? and (8) What perceptions do individuals have regarding MH and its treatment?

## Methods

### First Paper (Physicians)

The first paper will replicate the study performed by Dahlhausen et al [88] with adaptations. It will seek to understand the attitudes of physicians (both psychiatrists and psychologists) toward DMHA. The paper will allow us to characterize the implications of the supply side (ie, physicians who constitute the labor for the provision of MH services) for the adoption of DMHA in Portugal.

The study will be based on a cross-sectional, web-based survey. We will not include the qualitative part of Dahlhausen study as it was destined to complement the literature’s findings to develop a survey. Instead, we will take the final survey questionnaire made available on Dahlhausen’s study as given.

We will first translate it to Portuguese using a licensed translator and deliver it to physicians (both psychologists and psychiatrists, 5 each) for input. Physicians’ feedback will be focused on how to calibrate it to (1) reflect important questions to ask regarding the usage of digital health tools by doctors and (2) adapt to a Portuguese MH care context.

The feedback obtained from physicians will be incorporated to produce a final web-based survey questionnaire for this study. This survey will be deployed via Inqueritos@UP (the University of Porto’s internal survey manager, powered by LimeSurvey) as widely as possible and with the support of the Portuguese Order of Psychologists and the Psychiatry Specialty College of the Portuguese Order of Medical Doctors.

**Figure 1:**
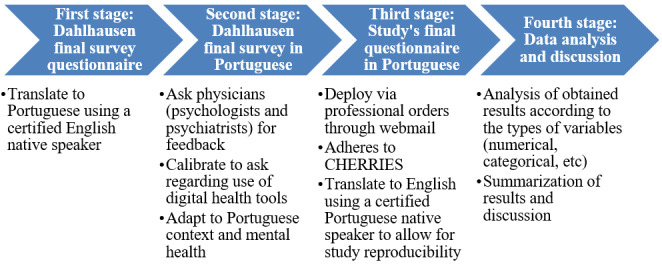
First study workflow. CHERRIES: Checklist for Reporting Results of Internet E-Survey.

The survey will adhere to and be reported following the CHERRIES (Checklist for Reporting Results of Internet E-Surveys) guidelines. For the certified translation of the Dahlhausen et al [88] questionnaire to Portuguese, as well as our final survey questionnaire in both Portuguese and English (the latter produced by a certified native Portuguese speaker translator from Portuguese to English), refer to Multimedia Appendix 1. Figure 1 summarizes the study’s stages.

There is no public information concerning the number of psychologists registered with the Order of Portuguese Psychologists. Registered psychiatrists (summing the stand-alone specialty with the subspecialty of children and adolescents) are a total of 1528. All will be invited to participate in this web-based survey via their respective professional orders.

To preserve privacy, respondents will not be asked to provide any identifiable information for completion of the survey. Additionally, invited participants will not be tracked for having started or completed the survey, increasing privacy but limiting the possibility of reminders. To establish a basis for comparison with a reimbursable apps system, we will ask physicians who answer the survey to bear in mind a scenario where these apps fulfill regulatory requirements and address safety, quality, and efficacy concerns. This establishes an equivalency between the definition of DiGA apps as outlined in the “Introduction” section and the definition of DMHA used in our study.

To maximize response, the only inclusion criterion will be that of being registered and of good standing (ie, not responsible for any infractions) with the physician’s respective professional order. No exclusion criteria will be introduced. There will be no financial incentives for filling the survey.

Gathered data will be assessed according to the same model as in the Dahlhausen et al [88] paper to allow for maximum comparability between results. This entails the breakdown between the sociodemographic characteristics of the sample and the answers to the questions in the survey concerning the 4 main topics at hand: perceived benefits, potential barriers to, measures to support adherence to, and prescription intentions for DMHA. All variables that allow for it will be characterized by descriptive statistical analysis. Estimates of association for the variables that correspond to the Dahlhausen et al [88] “Perceived Benefits From and Attitudes Toward DiGA” and “Prescription Intentions” subsections will also be computed.

Only data excluded due to different health system organizations and their consequences for citizens or physicians (eg, statutory health insurance in Germany vs little to no point-of-care payments in Portugal) or to reasonable suggestion during the foreseen feedback period will be treated differently, being subjected to either descriptive statistical analysis or association estimation according to the nature of the variable at hand.

### Second Paper (Individuals)

The second study produced in the context of this doctoral research project will replicate the paper by Borghouts et al [89]. The paper’s aim is to ascertain which factors determine public acceptance and usage of MH apps. The definition of MH apps in Borghouts study encompasses generic apps that can fall, for example, into the category of wellness, and our survey instrument will not impose any restrictions, thereby considering DMHA as an equivalent of MH apps in a broad sense.

The described study will simultaneously allow us to illustrate the demand side (ie, people who may potentially request MH services and care) for the adoption of MH apps in Portugal. Even though it is limited to an academic community, it is expected that it will have a reasonable mix of population characteristics. Limitations, both ex-ante and ex-post data collection, will be documented in the paper.

The study will start with a cross-sectional, web-based survey delivered through the University of Porto’s dynamic email function. The web-based survey will be based on the survey questionnaire made available in Borghouts paper. This questionnaire will be translated into Portuguese using a licensed translator. Afterward, it will be circulated between an equal number of students and teachers (estimated to be 5 each) affiliated with the Faculty of Medicine of the University of Porto for input. Received input will concentrate on how to adjust it to (1) include important questions to ask regarding the usage of digital health tools with a focus on MH and (2) adapt to a Portuguese MH context.

Obtained feedback will be incorporated to produce a final web-based survey questionnaire for this study. This survey will be deployed via Inqueritos@UP (the University of Porto’s internal survey manager, powered by LimeSurvey) as widely as possible and with the support of the University of Porto’s services.

**Figure 2:**
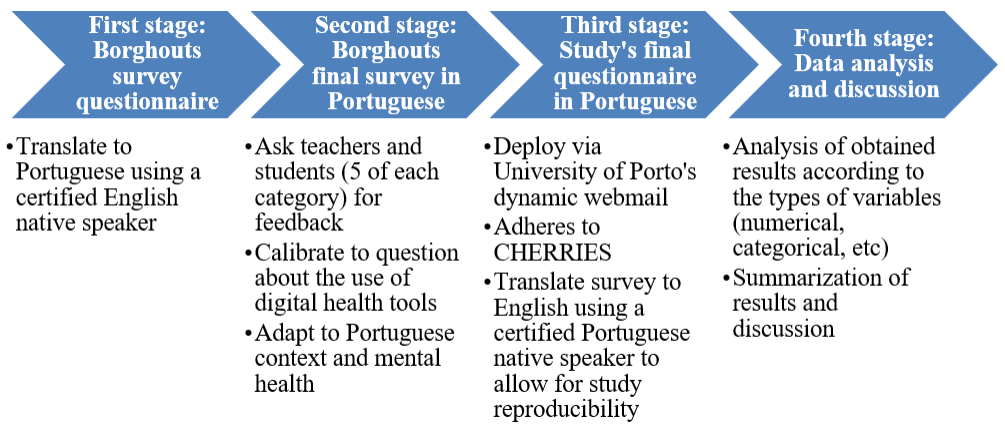
Second study workflow. CHERRIES: Checklist for Reporting Results of Internet E-Survey.

The survey will abide by and be reported following CHERRIES guidelines. For the certified translation of the Borghouts et al [89] questionnaire from English to Portuguese, as well as our final survey questionnaire in both Portuguese and English (the latter produced by a certified native Portuguese speaker translator from Portuguese to English), refer to Multimedia Appendix 2. Figure 2 summarizes the study’s stages.

It is estimated that the dynamic email function of the University of Porto will allow us to reach approximately 36,700 members of the University’s community and invite them to take part in this anonymous web-based survey. We will also ask the University of Porto’s website management team to divulge the survey, providing a link to access it and following all privacy-related issues.

To preserve privacy, respondents will not be asked to provide any identifiable information. Additionally, invited participants will not be tracked for having started or completed the survey, increasing privacy but limiting the possibility of reminders.

High comparability with the Borghouts et al [89] study is expected due to the nature of the questions in the Borghouts et al [89] survey instrument and their relevance in a Portuguese population, leading us to believe that few questions will be dropped or significantly altered. Gathered data will be assessed according to the same pattern as that offered in the Borghouts et al [89] paper to allow for maximum comparability between results. This means that, aside from a sociodemographic depiction of the sample, we will measure the same constructs as the Borghouts et al [89] paper: barriers to MH resources, important aspects of MH apps, activities people wish for in MH apps, the presence of MH issues, past use of professional MH services, perceived stress, perceived need to seek help, MH concerns, perceived stigma, the impact of other people’s expectations, and privacy concerns. Factor analysis and multicollinearity checking will be performed to limit the analysis to relevant constructs. Direct effect and full mediation models will be built.

All variables will be characterized by descriptive statistical analysis except when not possible. Only data that are excluded due to reasonable suggestion during pretest interviews will be treated differently, being subjected to either descriptive statistical analysis or association estimation according to the nature of the variable at hand.

To maximize response, the only inclusion criterion will be active registration with the University of Porto, defined as the ability to receive the email containing the invitation to answer the questionnaire. No exclusion criteria will be introduced. There will be no financial incentives for filling out the survey.

### Ethical Considerations

Ethical considerations and safeguards for the 2 manuscripts to be produced and their supporting documents include the following and incorporate advice received from the Data Protection Officer of the University of Porto:

- To preserve the privacy of participants, they will not be asked to provide any personally identifiable information. In addition, participants will not be tracked for having started or completed the survey, increasing privacy but limiting the possibility of reminders.
- Informed consent and consenting capacity: All potential participants (physicians and academic community members) will be given web-based written information on the study and its objectives and will be asked to provide consent (click-to-agree) that they are happy to participate, and that nonparticipation will not compromise their current roles. Participation in the study will be voluntary, and no inducements or incentives to participate will be offered.
- Confidentiality: Any data or personal details that could potentially reveal the identity of individuals will be removed. Only anonymized, deidentified information will leave the place of origin. A database with responses will be maintained on a password-protected database. All research data will be stored on a password-protected desktop computer at the host organization. Study participants will be invited, through a link provided on the last page of the survey, to provide their name and electronic address to allow the research team to facilitate their receiving a synopsis of the study findings on publication. This list will be kept separately on a password-protected database and a password-protected desktop computer at the host organization. All data will be stored securely at the host institution and destroyed 3 years after the PhD defense date.
- General Data Protection Regulation compliance will be adhered to in terms of the following:
  Data privacy rights: Participants will have the right to request information about their data throughout the research process.
  Transfer of data: Participants will be informed about the circumstances under which their data may be transferred and safety measures that will be taken to protect the data (eg, data are encoded).
  Retention of data: Participants will be informed how long their data will be stored.

By using Inqueritos@UP, survey data will be stored at the University of Porto’s servers, and thus not shared with external entities, constituting another layer of privacy protection.

The Ethics Committee of the Faculty of Medicine of the University of Porto has pronounced itself favorable to the research project on June 30, 2022.

### Ethics Approval

The research protocol was approved by the Doctoral Program in Health Data Science Director and the Ethics Committee of the Faculty of Medicine of the University of Porto (Rapport 52/CEFMUP/2022 of June 30, 2022). Data collection ran from September 26 and November 6, 2022, for the first study, and between September 20 and October 20, 2022, for the second study.

## Results

The findings of the proposed studies will elicit important information on how physicians and potential users perceive and respond to the usage of DMHAs to improve access to care and patient outcomes, along with the reduction of the burden of disease.

## Discussion

### Principal Findings

This will be the first research project known to the authors with the specific aim of assessing whether DMHA would be welcomed in Portugal and in a health care system with its political economy. It will do so by mapping the expectations of agents that determine the demand and supply sides for DMHA.

As such, the research project will provide valuable inputs on what would be the key success factors for any market- or government-based actors who decide to enter the market by collecting key information to advise health policy and service development in Portugal and in international settings by providing a key benchmarking tool by comparing Portugal with Germany (from the physicians’ point of view) and Portugal with the United States (from the individual’s point of view) in similar contexts.

It is also the first research project establishing a comparison between health systems with a very significant private sector component and systems where government plays a key role in ensuring access to and funding health and care, particularly concerning MH and the use of digital tools. Finally, in characterizing the market’s industrial organization by mapping the expectations of demand and supply sides, it adds to the literature on digital health economics.

### Strengths and Limitations

Potential limitations of the research project include bias associated with the method chosen to select the sample in the second paper, as it considers that the academic community replicates the country’s population distribution. Bias may impact external validity as this community may not necessarily replicate the Portuguese population’s digital literacy or access to information and communication technology tools, and therefore the implicit digital divide.

In addition, the sample’s average age may be younger than the country’s average due to the teacher-student ratio and the respective average ages of these groups. Lack of generalizability may also derive from participant self-selection; those more interested in research or in using digital health tools will more likely fill the survey. Question-order, interviewer, and acquiescence biases may have a minor influence on conducted surveys. Notwithstanding these limitations, our research project is expected to provide important information on physicians’ and individuals’ attitudes toward digital MH tools.

To counter the previously identified limitations and enhance the comparability of results, the authors have produced a “Checklist for a future researcher” (Multimedia Appendix 3). Its aim is to facilitate further research both in Portugal and abroad by listing the main points requiring adaptation to reproduce this protocol in different settings.

### Conclusions

The results of the studies projected in this research protocol will have implications for researchers and academia, industry, and policy makers concerning the adoption and implementation of digital health mental apps and associated interventions. Rapid and accessible screening, diagnostic, and treatment care strategies are needed to provide postpandemic access to MH care, and this project will inform key factors in designing and implementing successful digital health interventions based on DMHA both in Portugal and abroad.

## Appendix

Multimedia Appendix 1 First study’s final questionnaire in Portuguese and English.

Multimedia Appendix 2 Second study’s final questionnaire in Portuguese and English.

Multimedia Appendix 3 Checklist for a future researcher.

## Abbreviations

CHERRIES: Checklist for Reporting Results of Internet E-Surveys
DALY: disability-adjusted life year
DiGA: Digitale Gesundheitsanwendungen, in German, meaning digital health apps
DMHA: digital mental health app
INFARMED: Regulatory Authority for Medicines and Medical Devices (Autoridade Nacional do Medicamento e Produtos de Saúde, I.P.)
MH: mental health
NHS: National Health Service (Serviço Nacional de Saúde, or SNS, in Portuguese)
SPMS: Shared Services of the Portuguese Ministry of Health (Serviços Partilhados do Ministério da Saúde, in Portuguese)

## Data Availability

The data sets generated and analyzed during this study are not publicly available but are available from the corresponding author upon reasonable request.
