# Supplementary material for: Attitudes of Physicians and Individuals Toward Digital Mental Health Tools: Protocol for a Web-Based Survey Research Project
Source: JMIR Res Protoc. 2023 Mar 14;12:e41040. doi: 10.2196/41040 (PMC10131781; doi:10.2196/41040)
Supplement: Multimedia Appendix 1 [file resprot_v12i1e41040_app1.doc]

# Inquérito - Profissionais de Saúde Mental

*Quais as perceções dos profissionais de saúde mental sobre aplicações digitais de saúde? Quão digitalmente informados consideram estes profissionais que são, e quais as suas perceções sobre quão digitalmente letrados são os seus doentes/pacientes/utentes/clientes? Como é que os profissionais de saúde mental antecipam o seu próprio papel na transição digital dos cuidados de saúde, e quais os maiores benefícios, riscos, barreiras e oportunidades que consideram existir?*

No âmbito de uma investigação do Programa Doutoral em Ciência de Dados de Saúde na Faculdade de Medicina da Universidade do Porto, este estudo investiga as atitudes de psiquiatras e psicólogos em relação a aplicações digitais de saúde, incluindo aqueles que tenham tido pouco ou nenhum contacto prévio com aplicações digitais de saúde. O objetivo é ajudar os profissionais a lidar com esta nova forma de rastreio, diagnóstico, e terapêutica, assim como a determinar o nível de conhecimento dos profissionais de saúde mental. O preenchimento do questionário demorará 4-7 minutos.

*O que são as aplicações digitais de saúde?*

As aplicações digitais de saúde visam ajudar a rastrear, monitorizar, diagnosticar, e tratar ou minimizar sinais/sintomas de uma dada doença/perturbação.

*Existe enquadramento regulamentar para as aplicações digitais de saúde?*

Em Portugal as aplicações digitais de saúde estão sujeitas à atribuição do selo CE e, em alguns casos, ao Regulamento sobre Dispositivos Médicos, não existindo um percurso que efetue a sua validação para o acesso às tecnologias de saúde semelhante, por exemplo, ao dos medicamentos.

O programa DiGA (Digitale Gesundheitsanwendungen, ou “aplicações digitais de saúde” em alemão) foi implementado na Alemanha em outubro de 2020 com o objetivo de definir um procedimento claro e que permita a aprovação de aplicações digitais de saúde como dispositivos médicos e adicionalmente licenciadas pelo Instituto Federal de Medicamentos e Dispositivos Médicos (BfArM). As aplicações DiGA podem ser prescritas por médicos e são comparticipadas por todos os serviços públicos de saúde– como tal, também são denominadas “aplicações com prescrição médica”. A 1 de julho de 2022, 34 aplicações tinham recebido aprovação regulatória por este processo, sendo 14 destas consideradas como respondendo a patologias do foro psicológico/psiquiátrico. O sucesso deste programa até à data despoletou o interesse das autoridades de saúde francesas, que visam desenvolver um processo semelhante até ao final de 2022

Na Bélgica, o programa mHealthBelgium começou a efetuar avaliações e recomendações de reembolso em janeiro de 2021, após ter sido anunciado em 2018. Baseado numa avaliação constituída por uma pirâmide que representa três camadas de validação (assuntos legais e regulatórios, comunicação segura e privacidade, e financiamento e reembolso), este programa dispunha, a 2 de junho de 2022, de 36 aplicações digitais de saúde com algum nível de validação.

Pedimos-lhe assim que preencha este questionário utilizando primacialmente a sua própria experiência. De modo a permitir a comparabilidade máxima entre Portugal e a Alemanha (em que a utilização de aplicações digitais de saúde validadas é reembolsável) neste contexto, pedimos-lhe que responda às seguintes questões **assumindo**

**um cenário em que as aplicações digitais de saúde preenchem todos os requisitos regulatórios e são seguras, de qualidade, e eficazes**.

Este inquérito é realizado no âmbito de uma tese de doutoramento para o Programa Doutoral em Ciência de Dados de Saúde da FMUP, sendo a pessoa responsável pela recolha de dados o aluno Diogo Nogueira Leite (202002508), que pode ser contactado pelo endereço [up202002508@up.pt](mailto:up202002508@up.pt) para quais dúvidas que subsistam.

Existe(m) 27 questão(ões) neste questionário.

# Atitudes e perceções sobre aplicações digitais de saúde


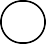


[Q1]

Qual o seu grau de concordância com a frase seguinte?

“*Sou a favor da possibilidade de médicos e psicólogos*

*prescreverem/recomendarem/utilizarem aplicações digitais de saúde clínica e tecnicamente validadas.*” *

Por favor, selecione **apenas uma** das seguintes opções:

Discordo totalmente Discordo

Não concordo nem discordo
 Concordo

Concordo totalmente


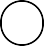

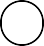


[Q3]

Como classifica a sua competência no que toca a... *

Por favor, selecione a posição apropriada para cada elemento:


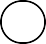


[Q2]

Qual o seu grau de concordância com a frase seguinte?

*“Sinto-me suficientemente informado sobre as aplicações digitais de saúde para as recomendar ou informar sobre estas.”* *

Por favor, selecione **apenas uma** das seguintes opções:

Discordo totalmente
 Discordo

Não concordo nem discordo

Concordo

Concordo totalmente

|  | **Muito má** | **Má** | **Nem boa nem má** | **Boa** | **Muito boa** |
| --- | --- | --- | --- | --- | --- |
| **...analisar o conjunto de aplicações digitais de saúde disponíveis?** | 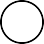 | 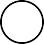 | 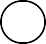 | 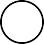 | 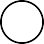 |
| **...informar os meus doentes/pacientes/**  **utentes/clientes/pessoas que me consultam**  **sobre aplicações digitais de saúde?** |  |  | 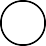 | 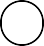 | 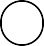 |
| **...distinguir entre as boas e as más aplicações digitais de saúde?** | 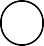 | 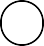 | 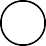 | 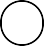 | 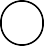 |


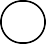


[Q4] Com que regularidade é que os seus doentes/pacientes/utentes/clientes o/a questionam sobre aplicações digitais de saúde em geral ou a propósito da sua prescrição? *

Por favor, selecione **apenas uma** das seguintes opções:

Nunca

Menos que uma vez por mês
 Mensalmente

Semanalmente
 Diariamente


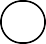


[Q5]

Qual o seu grau de concordância com a frase seguinte?

“*Os meus doentes/pacientes/utentes/clientes têm como expectativa que eu prescreva/aconselhe aplicações digitais de saúde.*” *

Por favor, selecione **apenas uma** das seguintes opções:

Discordo totalmente Discordo

Não concordo nem discordo
 Concordo

Concordo totalmente


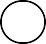


[Q6]

Com que regularidade informa sobre aplicações digitais de saúde? *

Por favor, selecione **apenas uma** das seguintes opções:

Nunca

Menos que uma vez por mês
 Mensalmente

Semanalmente

Diariamente


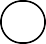


[Q7]

Qual a probabilidade de recomendar aplicações digitais de saúde nos próximos 12 meses? *

Por favor, selecione **apenas uma** das seguintes opções:

Muito improvável
 Improvável

Provável

Muito provável
 Sem opinião


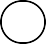


[Q8]

Qual o seu nível de concordância com a frase seguinte?

“*Tenho maior probabilidade de recomendar aplicações digitais de saúde a doentes/pacientes/utentes/clientes jovens*.” *

Por favor, selecione **apenas uma** das seguintes opções:

Discordo totalmente
 Discordo

Não concordo nem discordo

Concordo

Concordo totalmente


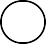


[Q9]

Qual o seu nível de concordância com a frase seguinte?

“*Tenho conhecimentos suficientes sobre cibersegurança, tratamento de dados pessoais, e outros elementos técnicos associados à minha atividade*

*atual.*” *

Por favor, selecione **apenas uma** das seguintes opções:

Não tenho conhecimentos deste tipo
 Muito baixo

Baixo

Médio
 Elevado

Muito elevado

## [Q10]

### Na sua opinião, quais os principais benefícios das aplicações digitais de saúde para os doentes/pacientes/utentes/clientes? *

Por favor, selecione a posição apropriada para cada elemento:

|  | **Discordo totalmente** | **Discordo** | **Não concordo**  **nem discordo** | **Concordo** | **Concordo totalmente** | **Não**  **sei** |
| --- | --- | --- | --- | --- | --- | --- |
| **Melhoria da capacidade de escolha informada dos doentes/pacientes/utentes/clientes, p. ex., opções adicionais de tratamento ou tratamento de acordo com guidelines/**  **diretrizes clínicas** | 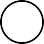 | 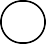 | 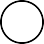 | 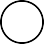 | 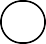 | 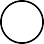 |
| **Adequada gestão da doença, p. ex., maior coordenação entre intervenientes nos processos de tratamento ou melhor gestão das dificuldades relacionadas com a doença no dia-a-dia** | 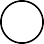 | 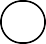 | 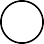 | 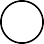 | 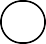 | 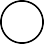 |
| **Maior adesão ao tratamento, p. ex., lembretes regulares sobre o tratamento acordado e motivação para a adoção de comportamentos saudáveis** | 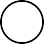 | 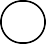 | 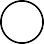 | 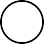 | 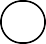 | 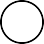 |

|  | **Discordo totalmente** | **Discordo** | **Não concordo**  **nem discordo** | **Concordo** | **Concordo totalmente** | **Não sei** |
| --- | --- | --- | --- | --- | --- | --- |
| **Melhor acesso a cuidados de saúde, p. ex., colmatando os tempos de espera para tratamento, oferecendo cuidados mais completos ou níveis mínimos de cuidados** | 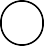 | 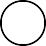 | 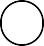 | 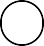 | 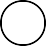 | 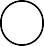 |
| **Maior literacia em saúde, p. ex., através de uma melhor educação da população** | 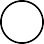 | 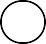 | 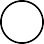 | 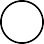 | 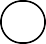 | 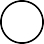 |

[Q10b]

Caso considere que existem outros benefícios das aplicações digitais de saúde para os doentes/pacientes/utentes/clientes que não foram listadas no quadro anterior, utilize o espaço abaixo para mencionar quais (opcional).

Por favor, escreva aqui a sua resposta:

## [Q11]

### Na sua opinião, quais as principais vantagens das aplicações digitais de saúde para os profissionais de saúde? *

Por favor, selecione a posição apropriada para cada elemento:

|  | **Discordo totalmente** | **Discordo** | **Não concordo**  **nem discordo** | **Concordo** | **Concordo totalmente** | **Não sei** |
| --- | --- | --- | --- | --- | --- | --- |
| **Satisfação de maior procura das mesmas por parte de novos doentes/pacientes/**  **utentes/clientes** | 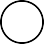 | 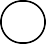 | 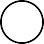 | 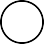 | 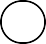 | 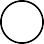 |
| **Maior satisfação dos doentes/pacientes/ utentes/clientes** | **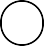** | **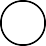** | 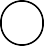 | 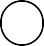 | 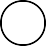 | 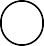 |
| **Economia de tempo por doente/paciente/**  **utente/cliente devido a ganhos de eficiência** | 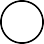 | 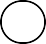 | 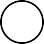 | 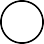 | 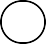 | 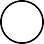 |
| **Melhor qualidade de assistência ao doente/paciente/**  **utente/cliente** | 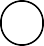 | 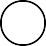 | 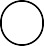 | 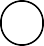 | 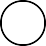 | 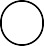 |
| **Maior sucesso no tratamento** | 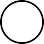 | 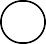 | 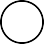 | 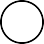 | 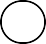 | 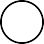 |
| **Opção de tratamento adicional** | 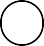 | 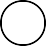 | 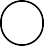 | 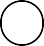 | 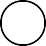 | 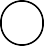 |

[Q11b] Caso considere que existem outros benefícios das aplicações digitais de saúde para os proﬁssionais de saúde que não foram listados no quadro anterior, utilize o espaço abaixo para mencionar quais (opcional).

Por favor, escreva aqui a sua resposta:

## [Q12]

### Quais considera serem as maiores barreiras/obstáculos à recomendação e/ou utilização de aplicações digitais de saúde? *

Por favor, selecione a posição apropriada para cada elemento:

|  | **Discordo totalmente** | **Discordo** | **Não concordo**  **nem discordo** | **Concordo** | **Concordo totalmente** | **Não sei** |
| --- | --- | --- | --- | --- | --- | --- |
| **Falta de informação sobre aplicações digitais de saúde, p. ex., sobre as aplicações disponíveis e sua adequação ao contexto clínico** | 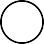 | 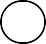 | 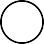 | 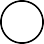 | 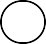 | 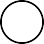 |
| **Falta de evidência científica ou evidência científica insuficiente sobre os benefícios para os pacientes** | 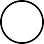 | 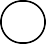 | 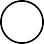 | 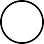 | 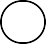 |  |
| **Nível de esforço inicial de formação e/ou familiarização para os profissionais de saúde** |  |  |  |  |  |  |
| **Necessidade de ajustes e adaptações dos processos de intervenção e/ou tratamento e/ou da prática clínica existentes** |  |  |  |  |  |  |

|  | **Discordo totalmente** | **Discordo** | **Não concordo**  **nem discordo** | **Concordo** | **Concordo totalmente** | **Não sei** |
| --- | --- | --- | --- | --- | --- | --- |
| **Fraca integração ou compatibilidade com software e ferramentas de prática clínica existente** |  |  |  |  |  |  |
| **Falta de apoio do fabricante em problemas ou questões técnicas** |  |  |  |  |  |  |
| **Aumento permanente do volume de trabalho de médicos e psicólogos devido a tarefas adicionais na assistência aos pacientes** |  |  |  |  |  |  |
| **Ausência de definição sobre mecanismos de comparticipação na utilização de aplicações digitais de saúde ou serviços médicos/terapêuticos de acompanhamento requeridos pelas mesmas** |  |  |  |  |  |  |
| **Incerteza quanto à privacidade e segurança dos dados pessoais (de saúde)** |  |  |  |  |  |  |

|  | **Discordo totalmente** | **Discordo** | **Não concordo**  **nem discordo** | **Concordo** | **Concordo totalmente** | **Não sei** |
| --- | --- | --- | --- | --- | --- | --- |
| **Incerteza quanto a questões de consentimento informado (p. ex., capacidade de retirada do consentimento)** |  |  |  |  |  |  |
| **Outras questões éticas e ambiguidades legais,**  **p. ex., em relação ao risco de responsabilidade em caso de diagnóstico ou tratamento incorreto com base em dados da aplicação** |  |  |  |  |  |  |

[Q12b] Caso considere que existem outras barreiras/obstáculos à recomendação e/ou utilização de aplicações digitais de saúde para os doentes/pacientes/utentes/clientes que não

foram listadas no quadro anterior, por favor utilize o espaço abaixo para mencionar quais (opcional)

Por favor, escreva aqui a sua resposta:

## [Q13]

### O que é que poderá incentivá-lo/a a recomendar e/ou utilizar aplicações digitais de saúde? *

Por favor, selecione a posição apropriada para cada elemento:

|  | **Discordo totalmente** | **Discordo** | **Não concordo**  **nem discordo** | **Concordo** | **Concordo totalmente** | **Não sei** |
| --- | --- | --- | --- | --- | --- | --- |
| **Mais informação sobre as aplicações disponíveis e sua adequação ao objetivo de saúde subjacente (rastreio, tratamento, descrição por patologia, etc.)** |  |  |  |  |  |  |
| **Definição de processo de prescrição / diretrizes de aconselhamento e utilização** |  |  |  |  |  |  |
| **Alteração de enquadramento legislativo sobre as opções de rastreio, diagnóstico, tratamento e prognóstico passíveis de serem recomendadas e/ou utilizadas por profissionais de saúde** |  |  |  |  |  |  |
| **Existência de evidência científica da validade das aplicações enquanto intervenção de saúde** |  |  |  |  |  |  |

|  | **Discordo totalmente** | **Discordo** | **Não concordo**  **nem discordo** | **Concordo** | **Concordo totalmente** | **Não sei** |
| --- | --- | --- | --- | --- | --- | --- |
| **Recomendação de aplicações específicas por instituições de saúde (DGS, INFARMED, EMA, etc.)** |  |  |  |  |  |  |
| **Integração de aplicações como parte dos pacotes comerciais providenciados por seguros de saúde** |  |  |  |  |  |  |
| **Questões levantadas ou pedidos por parte dos próprios doentes/pacientes/**  **utentes/clientes** |  |  |  |  |  |  |
| **Linha de apoio ao profissional de saúde providenciada pelo fabricante/**  **desenvolvedor de uma aplicação**  **digital de saúde** |  |  |  |  |  |  |
| **Relatos/partilha de experiências positivas de colegas, p. ex., em congressos ou em revistas especializadas** |  |  |  |  |  |  |
| **Recomendação de uma ou mais aplicações por sociedades profissionais/diretrizes clínicas específicas** |  |  |  |  |  |  |

[Q13b] Caso considere que existem outros fatores que possam incentivá-lo a prescrever aplicações digitais de saúde que não foram listadas no quadro anterior, por favor utilize o espaço abaixo para mencionar quais (opcional)

Por favor, escreva aqui a sua resposta:

### [Q14] Indique quais as informações sobre as aplicações digitais de saúde que lhe

interessariam em caso de ser possível, no panorama português, prescrevê-las/recomendá-las? (possibilidade de múltipla seleção) *

Por favor, selecione **todas** as que se aplicam:

Quais as aplicações existentes?

Para que patologias ou outras indicações é que uma aplicação é adequada? Quais os benefícios e riscos comprovados de uma aplicação?

Quanto custa uma aplicação digital de saúde? O SNS e/ou as companhias de seguros de saúde comparticipam-na? De que forma?

Como é que prescrevo/recomendo a utilização de uma aplicação?

Como é que os meus doentes/pacientes/utentes/clientes obtêm uma aplicação depois de eu a prescrever/recomendar, e como é que a devem utilizar?

Como é que monitorizo a utilização de uma aplicação?

Não desejo receber qualquer informação adicional sobre as aplicações. Outro:

[Q15] Por favor, explique mais aprofundadamente a sua opinião sobre as aplicações digitais de saúde (opcional).

Por favor, escreva aqui a sua resposta:

# Questões sociodemográficas

[Q01]

Qual destas carateriza a sua profissão? *

Por favor, selecione **apenas uma** das seguintes opções:

Psicólogo/a

Psiquiatra da infância e da adolescência
 Psiquiatra de adultos

Outro

[Q02] Trabalha numa área geodemográfica com *

Por favor, selecione **apenas uma** das seguintes opções:

Mais de 500.000 habitantes

100.001 – 500.000 habitantes

20.001 – 100.000 habitantes

5.001 – 20.000 habitantes
 Menos de 5.000 habitantes

[Q03]

Que tipo de local de trabalho melhor descreve a sua atual situação profissional? (selecione todas as aplicáveis) *

Por favor, selecione **todas** as que se aplicam:

Hospital

Cuidados de saúde primários Clínica

Consultório individual (sem outros colegas)

Consultório com outros colegas (p. ex., consultório conjunto ou de grupo)

Outro:

[Q04]

Trabalha no Serviço Nacional de Saúde (SNS), no setor privado, ou em ambos? *

Por favor, selecione **apenas uma** das seguintes opções:

Apenas no SNS

Apenas no setor privado
 Em ambos

[Q05] Quantos médicos ou psicólogos (cf. o caso) trabalham no seu local de trabalho?

Por favor, escreva aqui a sua resposta:

[Q06]

Qual o seu nível de interação digital em contexto profissional, numa escala de 1 (sem qualquer interação digital) a 10 (com muita interação digital)?

*

 A resposta deve estar entre 1 e 10

 Neste campo apenas pode ser introduzido um valor inteiro. Por favor, escreva aqui a sua resposta:

[Q07] Em que faixa etária se encontra a sua idade? *

Por favor, selecione **apenas uma** das seguintes opções:

Menos de 26 anos
 26 – 35 anos

36 – 45 anos

46 – 55 anos

56 – 65 anos
 Mais de 65 anos

[Q08]

Qual o seu género? *

Por favor, selecione **apenas uma** das seguintes opções:

Feminino
 Masculino

Outro

Caso deseje ser contactado(a) para o recebimento de uma sinopse dos resultados decorrentes [deste inquérito em estudo publicado, por favor preencha este formulário (https://inqueritos.up.pt/index.php?r=survey/index&sid=489829&lang=pt)](https://inqueritos.up.pt/index.php?r=survey/index&sid=489829&lang=pt).

Obrigado pela sua participação e contribuição para a investigação!

Obrigado por ter concluído este inquérito.

# Survey - Mental Health Professionals

*What are mental health professionals' perceptions of digital health apps? How digitally informed do these professionals consider themselves to be, and what are their perceptions of how digitally literate are their patients/users/clients? How do mental health professionals anticipate their own role in the digital transition of healthcare, and what do they see as the greatest benefits, risks, challenges and opportunities?*

Within the framework of a research carried out in the PhD Programme in Health Data Science at the Faculty of Medicine of the University of Porto, this study investigates the attitudes of psychiatrists and psychologists towards digital health apps, including those who have had little or no previous contact with digital health apps. The goal is to help professionals deal with this new screening, diagnostic, and therapeutic approach, as well as to determine the level of knowledge of mental health professionals. Completing the survey will take 4-7 minutes.

*What are digital health apps?*

Digital health apps were designed to help track, monitor, diagnose, and treat or minimise signs/symptoms of a given disease/disorder.

*Is there a regulatory framework for digital health apps?*

In Portugal, digital health apps are subject to the CE marking and, in some cases, to the Medical Devices Regulation, and it is not possible to validate them for access to health technologies such as medicines.

The DiGA programme (Digitale Gesundheitsanwendungen, or "digital health apps" in German) was implemented in Germany in October 2020 in order to define a clear procedure that enables digital health apps to be approved as medical devices and additionally licensed by the Federal Institute for Medicines and Medical Devices (BfArM). DiGA apps can be prescribed by doctors and are reimbursed by all public health services - as such they are also called "medical prescription apps". As of July 1^st^, 2022, 34 apps had received regulatory approval through this process, and 14 of these were considered to respond to psychological/psychiatric pathologies. The success of this programme to this date has triggered interest from French health authorities, who aim to develop a similar process by the end of 2022.

In Belgium, the mHealthBelgium programme started making assessments and reimbursement recommendations in January 2021, after being announced in 2018. Based on an assessment consisting of a pyramid representing three stages of validation (legal and regulatory issues, secure communication and privacy, and financing and reimbursement), this programme had 36 digital health apps with some level of validation as of June 2^nd^, 2022.

We therefore ask you to complete this survey drawing mainly from your own experience. In order to guarantee maximum comparability between Portugal and Germany (where the use of validated digital health apps is reimbursable) in this context, we ask you to answer the following questions **assuming digital health apps meet all regulatory requirements and are safe, of reliable quality, and effective**.

This survey is being conducted as part of a PhD thesis for the PhD Programme in Health Data Science at the FMUP. The person responsible for collecting data is the student Diogo Nogueira Leite (202002508), who can be contacted at up202002508@up.pt for further questions.

This survey is comprised of 27 question(s).

# Attitudes and perceptions about digital health apps

[Q1]

Please indicate your level of agreement with the following sentence:

“*I am in favour of allowing doctors and psychologists to prescribe/recommend/use technically validated clinical digital health apps."* *

Please select **only one** of the following options:

Strongly disagree

Disagree

Neither agree nor disagree
 Agree

Strongly agree

[Q3]

How would you rate your ability when it comes to... *

Please select the appropriate position for each element:

[Q2]

Please indicate your level of agreement with the following sentence:

*"I feel sufficiently informed about digital health apps to recommend or inform about them."* *

Please select **only one** of the following options:

Strongly disagree
 Disagree

Neither agree nor disagree

Agree

Strongly agree

|  | **Very**  **bad** | **Bad** | **Neither good**  **nor bad** | **Good** | **Very**  **good** |
| --- | --- | --- | --- | --- | --- |
| **...analyse the range of digital health applications available?** |  |  |  |  |  |
| **...giving my patients/users/clients/**  **people who consult me information about digital health apps?** |  |  |  |  |  |
| **...distinguish between good and bad digital health apps?** |  |  |  |  |  |

[Q4] How regularly do your patients/users/clients ask you about digital health apps in general or about your prescription? *

Please select **only one** of the following options:

Never

Less than once a month
 On a monthly basis

On a weekly basis
 On a daily basis

[Q5]

Please indicate your level of agreement with the following sentence:

“*My patients/users/clients expect that I will prescribe/advise digital health apps." **

Please select **only one** of the following options:

Strongly disagree

Disagree

Neither agree nor disagree
 Agree

Strongly agree

[Q6]

How regularly do you give information about digital health applications? *

Please select **only one** of the following options:

Never

Less than once a month
 On a monthly basis

On a weekly basis
 On a daily basis

[Q7]

How likely are you to recommend digital health apps in the next 12 months? *

Please select **only one** of the following options:

Very unlikely
 Unlikely

Likely

Very likely
 No opinion

[Q8]

Please indicate your level of agreement with the following sentence:

“*I am more likely to recommend digital health apps to young patients/users/clients."* *

Please select **only one** of the following options:

Strongly disagree
 Disagree

Neither agree nor disagree
 Agree

Strongly agree

[Q9]

Please indicate your level of agreement with the following sentence:

“*I have sufficient knowledge of cybersecurity, processing of personal data, and other technical elements associated with my current activity."* *

Please select **only one** of the following options:

I have no knowledge of this kind
 Very low

Low

Medium
 High

Very high

## [Q10]

### In your opinion, what are the main benefits of digital health apps for patients/users/clients? *

Please select the appropriate position for each element:

|  | **Strongly disagree** | **Disagree** | **Neither agree nor disagree** | **Agree** | **Strongly agree** | **I don’t know** |
| --- | --- | --- | --- | --- | --- | --- |
| **Improved ability of patients/ /users/clients to make informed choices, e.g. additional treatment options or treatment according to clinical guidelines** |  |  |  |  |  |  |
| **Proper disease management, e.g. increased coordination between all those involved in treatment processes or better management of disease-related difficulties in daily life** |  |  |  |  |  |  |
| **Wider adherence to treatment e.g. regular reminders about agreed treatment and motivation to adopt healthy behaviours** |  |  |  |  |  |  |

|  | **Strongly disagree** | **Disagree** | **Neither agree nor disagree** | **Agree** | **Strongly agree** | **I don’t know** |
| --- | --- | --- | --- | --- | --- | --- |
| **Improved access to healthcare, e.g. bridging the gap between waiting times for treatment, offering more comprehensive care or minimum levels of care** |  |  |  |  |  |  |
| **Increased health literacy, e.g. through better education of the population** |  |  |  |  |  |  |

[Q10b]

If you think there are other benefits of digital health apps for patients/users/clients that have not been listed in the previous table, please use the space below to mention which ones (optional).

Please write your answer here:

## [Q11]

### In your opinion, what are the main advantages of digital health apps for healthcare professionals? *

Please select the appropriate position for each element:

|  | **Strongly disagree** | **Disagree** | **Neither agree nor disagree** | **Agree** | **Strongly agree** | **I don’t know** |
| --- | --- | --- | --- | --- | --- | --- |
| **Satisfaction resulting from a greater demand from new patients/users/clients** |  |  |  |  |  |  |
| **Increased patient/user/client satisfaction** |  |  |  |  |  |  |
| **Time savings per patient/user/client due to efficiency gains** |  |  |  |  |  |  |
| **Better quality of care for the patient/**  **user/client** |  |  |  |  |  |  |
| **Greater treatment success** |  |  |  |  |  |  |
| **Additional treatment option** |  |  |  |  |  |  |

[Q11b] If you think there are other benefits of digital health apps for healthcare professionals that were not listed in the previous table, please use the space below to mention which ones (optional).

Please write your answer here:

## [Q12]

### What do you consider to be the biggest challenges/obstacles to the recommendation and/or use of digital health apps? *

Please select the appropriate position for each element:

|  | **Strongly disagree** | **Disagree** | **Neither agree nor disagree** | **Agree** | **Strongly agree** | **I don’t know** |
| --- | --- | --- | --- | --- | --- | --- |
| **Lack of information on digital health apps, e.g. on available apps and their suitability in the clinical setting** |  |  |  |  |  |  |
| **Lack of scientific evidence or insufficient scientific evidence on the benefits for patients** |  |  |  |  |  |  |
| **Level of initial training and/or familiarisation effort for health professionals** |  |  |  |  |  |  |
| **Need for adjustments and adaptations of existing intervention and/or treatment processes and/or clinical practice** |  |  |  |  |  |  |

|  | **Strongly disagree** | **Disagree** | **Neither agree nor disagree** | **Agree** | **Strongly agree** | **I don’t know** |
| --- | --- | --- | --- | --- | --- | --- |
| **Poor integration or compatibility with existing software and clinical practice tools** |  |  |  |  |  |  |
| **Lack of support from the manufacturer on problems or technical issues** |  |  |  |  |  |  |
| **Permanent increase in the workload of doctors and psychologists due to additional tasks in patient care** |  |  |  |  |  |  |
| **Absence of definition on co-payment mechanisms for the use of digital health apps or accompanying medical/therapeutic services required by them** |  |  |  |  |  |  |
| **Uncertainty about privacy and security of personal (health) data** |  |  |  |  |  |  |

|  | **Strongly disagree** | **Disagree** | **Neither agree nor disagree** | **Agree** | **Strongly agree** | **I don’t know** |
| --- | --- | --- | --- | --- | --- | --- |
| **Uncertainty about informed consent issues (e.g. ability to withdraw consent)** |  |  |  |  |  |  |
| **Other ethical issues and legal ambiguities,**  **e.g. in relation to the risk of liability in case of incorrect diagnosis or treatment based on data from the app** |  |  |  |  |  |  |

[Q12b] If you think there are other challenges/obstacles to the recommendation and/or use of digital health apps for patients/users/clients that were listed in the previous table, please use the space below to mention which ones (optional)

Please write your answer here:

## [Q13]

### What might encourage you to recommend and/or use digital health apps? *

Please select the appropriate position for each element:

|  | **Strongly disagree** | **Disagree** | **Neither agree nor disagree** | **Agree** | **Strongly agree** | **I don’t know** |
| --- | --- | --- | --- | --- | --- | --- |
| **More information on the available apps and their suitability for the underlying health objective (screening, treatment, description by pathology, etc.)** |  |  |  |  |  |  |
| **Definition of prescribing process / advice and use guidelines** |  |  |  |  |  |  |
| **Legislative framework change on screening, diagnosis, treatment and prognosis options that may be recommended and/or used by healthcare professionals** |  |  |  |  |  |  |
| **Existence of scientific evidence of the validity of the apps as a health intervention** |  |  |  |  |  |  |

|  | **Strongly disagree** | **Disagree** | **Neither agree nor disagree** | **Agree** | **Strongly agree** | **I don’t know** |
| --- | --- | --- | --- | --- | --- | --- |
| **Recommendation of specific apps by health institutions (DGS, INFARMED, EMA, etc.)** |  |  |  |  |  |  |
| **Integration of apps as part of the commercial packages provided by health insurers** |  |  |  |  |  |  |
| **Issues raised or requests made by patients/clients/**  **users themselves** |  |  |  |  |  |  |
| **Helpline provided by the manufacturer/**  **developer of a digital health app** |  |  |  |  |  |  |
| **Reporting/sharing positive experiences of colleagues, e.g. at conferences or in peer-reviewed journals** |  |  |  |  |  |  |
| **Recommendation of one or more apps by specific clinical guidelines/**  **professional societies** |  |  |  |  |  |  |

[Q13b] If you think there are other factors that might encourage you to prescribe digital health apps that were not listed in the previous table, please use the space below to mention which ones (optional)

Please write your answer here:

### [Q14] Please indicate which information on digital health apps you would be interested in if it were possible, in the Portuguese context, to prescribe/recommend them? *

Please select **all** that apply:

What apps are there?

For which pathologies or other indications is an app suitable? What are the proven benefits and risks of an app?

How much does a digital health app cost? Does the National Health Service and/or health insurance companies pay for it? In what way?

How do I prescribe/recommend the use of an app?

How do my patients/users/clients get an app after I prescribe/recommend it, and how should they use it?

How do I monitor the use of an app?

I do not wish to receive any further information about apps.

Other:

[Q15] Please further develop your views on digital health apps (optional).

Please write your answer here:

# Sociodemographic questions

[Q01]

Which of these describes your occupation? *

Please select **only one** of the following options:

Psychologist

Child and adolescent psychiatrist
 Adult psychiatrist

Other

[Q02] You work in a geodemographic area with *

Please select **only one** of the following options:

More than 500,000 inhabitants

100,001 – 500,000 inhabitants

20,001 – 100,000 inhabitants

5,001 – 20,000 inhabitants
 Less than 5,000 inhabitants

[Q03]

Which type of workplace best describes your current occupational status? (select all that apply) *

Please select **all** that apply:

Hospital

Primary health care Clinic

Private practice (no other colleagues)

Practice with other colleagues (e.g., joint or group practice)

Other:

[Q04]

Do you work in the National Health Service (SNS), in the private sector, or in both? *

Please select **only one** of the following options:

Only in SNS

Only in the private sector
 In both

[Q05] How many doctors or psychologists (cf. the case) work at your workplace?

Please write your answer here:

[Q06]

What is your level of digital interaction in a professional context, on a scale of 1 (no digital interaction at all) to 10 (a lot of digital interaction)? *

 The answer must be between 1 and 10

 You can only enter a whole number in this field.

Please write your answer here:

[Q07] What is your age group? *

Please select **only one** of the following options:

Under 26 years old
 26 – 35 years old

36 – 45 years old

46 – 55 years old

56 – 65 years old
 Over 65 years old

[Q08]

What gender do you identify as? *

Please select **only one** of the following options:

Female
 Male

Other

Should you wish to be contacted to receive a summary of the results arising from this survey in a published study, please complete this form (https://inqueritos.up.pt/index.php?r=survey/index&sid=489829&lang=pt).

Thank you for your participation and contribution to the research!

Thank you for completing this survey.
