# Supplementary material for: Attitudes of Physicians and Individuals Toward Digital Mental Health Tools: Protocol for a Web-Based Survey Research Project
Source: JMIR Res Protoc. 2023 Mar 14;12:e41040. doi: 10.2196/41040 (PMC10131781; doi:10.2196/41040)
Supplement: Multimedia Appendix 2 [file resprot_v12i1e41040_app2.docx]

Inquérito - Comunidade Académica

**Questionário sobre a forma como as Tecnologias Móveis Podem Satisfazer as Necessidades Não Satisfeitas de Saúde Mental e de Bem-estar da Comunidade Académica da Universidade do Porto**

A finalidade deste questionário é compreender as necessidades não satisfeitas de saúde mental da comunidade académica (i.e., estudantes, professores e funcionários) e as estratégias que usam atualmente para lidar com a sua saúde mental, com vista a providenciar os recursos adequados (incluindo ferramentas digitais). Investigações prévias revelaram que os estudantes universitários se deparam com problemas de saúde mental e de falta de bem-estar que muitas vezes não são identificados e intervencionados, e que é necessário um melhor acesso a recursos dirigidos à saúde mental. No entanto, falta uma investigação sobre as necessidades de saúde mental dos estudantes universitários e sobre a disponibilidade e utilização da tecnologia para satisfazer essas necessidades. Bem assim, não existe um estudo que aborde, para além das necessidades não satisfeitas dos estudantes, aquelas dos professores e dos funcionários. Embora a literatura seja consensual no que respeita a um maior risco de transtornos do foro psicológico e emocional entre adolescentes e jovens adultos, não se pode construir um retrato completo da comunidade académica sem contar com estes outros dois grupos. Tal englobamento tem igualmente a vantagem de gerar um entendimento que permita uma aproximação à realidade da população portuguesa, acautelando potenciais vieses.

Pretendemos investigar as seguintes questões:

1. Quais as atuais necessidades não satisfeitas de saúde mental e bem-estar da comunidade académica?
2. Quais as atuais ferramentas e estratégias usadas para lidar com a saúde e o bem-estar?
3. Como é que as ferramentas digitais, como as aplicações móveis, podem satisfazer necessidades não satisfeitas?
4. Que fatores poderão influenciar a adoção/envolvimento de aplicações móveis de saúde mental?

Este inquérito é realizado no âmbito de uma tese de doutoramento para o Programa Doutoral em Ciência de Dados de Saúde da FMUP, sendo a pessoa responsável pela recolha de dados o aluno Diogo Nogueira Leite (202002508), que pode ser contactado pelo endereço [up202002508@up.pt](mailto:up202002508@up.pt) para quais dúvidas que subsistam.

Existe(m) 60 questão(ões) neste questionário.

# Informação demográfica e de base

Esta parte do questionário é sobre a sua informação básica e demográfica. Estes dados servem exclusivamente para caraterização da amostra das respostas e não serão usados individualmente ou para outros fins que não os de caracterização global.Esta parte do questionário é sobre a sua informação básica e demográfica. Estes dados servem exclusivamente para caraterização da amostra das respostas e não serão usados individualmente ou para outros fins que não os de caracterização global.

[Q1] Qual a sua idade? (Coloque apenas o número de anos de idade que tem. Caso não deseje responder, por favor insira 0) *

Por favor, escreva aqui a sua resposta:


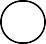


[Q2]

Qual o seu género? *

Por favor, selecione **apenas uma** das seguintes opções:

Homem
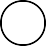
 Mulher


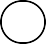
 Homem transgénero


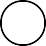
 Mulher transgénero


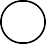
 Genderqueer/ Não conforme / Não-binário
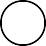
 Sem certezas quanto à identidade de género
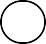
 Prefiro não responder.


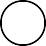
 Outro

[Q3]

Selecione a opção que melhor descreve a sua situação. (Selecione as respostas aplicáveis.) *

Por favor, selecione **todas** as que se aplicam:

Estudante a tempo inteiro de primeiro ciclo de estudos (licenciatura) Estudante a tempo inteiro de segundo ciclo (mestrado)

Estudante a tempo inteiro de ensino pós-graduado/especialização Estudante a tempo inteiro de terceiro ciclo (doutoramento) Estudante a tempo parcial de primeiro ciclo

Estudante a tempo parcial de segundo ciclo

Estudante a tempo parcial de ensino pós-graduado/especialização Estudante a tempo parcial de terceiro ciclo

Professor(a) (independentemente da categoria profissional) Funcionário(a) da U. Porto e/ou suas Unidades Orgânicas Prefiro não responder.

Outro:


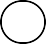


[Q4] Por favor, selecione a opção que melhor descreve a sua situação de emprego. *

Por favor, selecione **apenas uma** das seguintes opções:

Empregado(a) a tempo inteiro
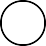
 Empregado(a) a tempo parcial


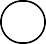
 Estudante a tempo inteiro (inclui bolseiros)


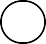
 Trabalhador(a) estudante


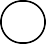
 Desempregado(a) à procura de emprego


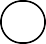
 Desempregado(a) que não está à procura de emprego
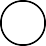
 Prefiro não responder


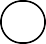
 Outro


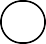


[Q5]

Qual o horário em que frequenta a maioria das aulas na Universidade do Porto? (Caso não seja estudante ou se encontre, p. ex., em elaboração de tese sem componente curricular, por

favor assinale “Não aplicável”) *

Por favor, selecione **apenas uma** das seguintes opções:

Diurno


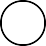
 Pós-laboral
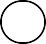
 Não aplicável


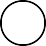
 Prefiro não responder
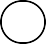
 Outro


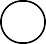


[Q6]

Qual a sua etnia? (Selecione uma resposta. Se tiver várias etnias, selecione a opção “mais do que uma etnia”. Categorização baseada nos Censos 2021) *

Por favor, selecione **apenas uma** das seguintes opções:

Branco(a)/Português(a) branco(a)/De origem europeia


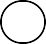
 Negro(a)/Português(a) negro(a)/Afrodescendente/De origem africana
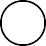
 Asiático(a)/Português(a) de origem asiática/De origem asiática


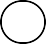
 Cigano/Português cigano/Roma
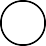
 Mais do que uma etnia


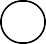
 Prefiro não responder.


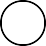
 Outro


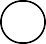


[Q7]

Por favor, selecione a opção que melhor descreve o seu atual estado civil. *

Por favor, selecione **apenas uma** das seguintes opções:


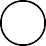
 Solteiro(a)

Numa relação de compromisso, mas não casado(a)
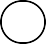
 Casado(a)


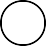
 Viúvo(a)


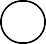
 Divorciado(a)
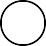
 Separado(a)


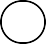
 Prefiro não responder.


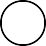
 Outro


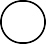


[Q8]

Tem filhos ou dependentes? *

Por favor, selecione **apenas uma** das seguintes opções:

Sim
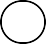
 Não


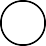
 Prefiro não responder.


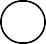


[Q9]

Qual a sua atual situação de habitação? (Selecione uma resposta – caso tenha dúvidas, considere a situação em que vive mais tempo.) *

Por favor, selecione **apenas uma** das seguintes opções:

Vivo sozinho(a)


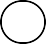
 Vivo com o(a) parceiro(a) ou cônjuge
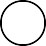
 Vivo com colegas de casa


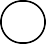
 Vivo com a minha família
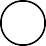
 Prefiro não responder


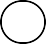
 Outro


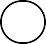


[Q10]

Está atualmente em situação de sem-abrigo? *

Por favor, selecione **apenas uma** das seguintes opções:


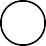
 Sim Não


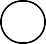
 Prefiro não responder.


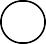


[Q11]

Qual o atual rendimento anual do seu agregado familiar?

*

Por favor, selecione **apenas uma** das seguintes opções:

Inferior a 5.000€ (ca. 416€/mês)
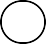
 5.001-10.000€ (417-833€/mês)


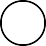
 10.001€ – 13.500€ (834-1.125€/mês)


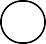
 13.501€ – 19.000€ (1.126-1.583€/mês)


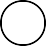
 19.001€ – 27.500€ (1.584-2.291€/mês)


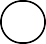
 27.501€ – 32.500€ (2.292-2.708€/mês)


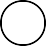
 32.501€ – 40.000€ (2.709-3.333€/mês)


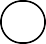
 40.001€ – 50.000€ (3.334-4.166€/mês)


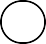
 50.001€ – 100.000€ (4.167-8.333€/mês)


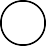
 100.001€ – 250.000€ (8.334-20.833€/mês)


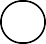
 Superior a 250.000€ (> 20.833€/mês)
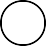
 Não sei


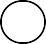
 Prefiro não responder


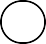


[Q12]

Tem alguma incapacidade?

Para este questionário, a incapacidade define-se como um problema de saúde mental ou

físico que dure há mais de 6 meses e que limite as principais atividades vitais, mas que não seja resultado de uma doença mental grave. *

Por favor, selecione **apenas uma** das seguintes opções:


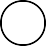
 Sim Não


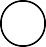
 Prefiro não responder.


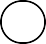


[Q12a]

Que tipo de incapacidade tem? (Selecione todas as opções aplicáveis.)

Para este questionário, a incapacidade define-se como um problema de saúde mental ou física

que dure há mais de 6 meses e que limite as principais atividades vitais, mas que não seja resultado de uma doença mental grave. *

Responda a esta pergunta apenas se as seguintes condições são verdadeiras:

A resposta for 'Sim' na pergunta '12 [Q12]' (Tem alguma incapacidade? Para este questionário, a incapacidade define-se como um problema de saúde mental ou físico que dure há mais de 6 meses e que limite as principais atividades vitais, mas que não seja resultado de uma doença mental grave.)

Por favor, selecione **apenas uma** das seguintes opções:

Incapacidade mental ligeira a moderada
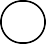
 Incapacidade física / de mobilidade


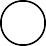
 Problema de saúde crónico (incluindo dor crónica, patologia cancerígena, patologia

neurológica)


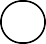
 Incapacidade visual
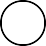
 Incapacidade auditiva
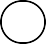
 Prefiro não responder.


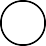
 Outro

# Utilização de tecnologia em geral e do smartphone

Esta secção é sobre a sua utilização de tecnologia e do smartphone.

[Q13] Qual ou quais dos seguintes dispositivos usa? (Selecione todas as opções aplicáveis.) *

 Selecione todas as opções que se apliquem Por favor, selecione **todas** as que se aplicam:

Computador (portátil ou de secretária) Smartphone

Tablet

Telemóvel, mas não um smartphone

Wearables (smartwatches, pulseiras de atividade, etc.) Não uso nenhuma das opções anteriores.

Prefiro não responder.

### [Q13a]


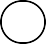

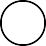

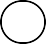

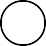

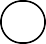

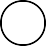


Num dia normal, com que regularidade usa um computador ou dispositivo móvel para aceder às redes sociais?

*As redes sociais podem incluir Facebook, Instagram, Twitter, Snapchat, LinkedIn, TikTok, Pinterest, Telegram, WeChat, Weibo, etc.* *

Responda a esta pergunta apenas se as seguintes condições são verdadeiras:

-------- Scenario 1 --------

A resposta for na pergunta '14 [Q13]' (Qual ou quais dos seguintes dispositivos usa? (Selecione todas as opções aplicáveis.))

-------- ou Scenario 2 --------

A resposta for na pergunta '14 [Q13]' (Qual ou quais dos seguintes dispositivos usa? (Selecione todas as opções aplicáveis.))

-------- ou Scenario 3 --------

A resposta for na pergunta '14 [Q13]' (Qual ou quais dos seguintes dispositivos usa? (Selecione todas as opções aplicáveis.))

 Escolher uma das seguintes respostas

Por favor, selecione **apenas uma** das seguintes opções:

Quase constantemente (em média, mais de 6 horas por dia) Muitas vezes por dia (em média, entre 4 e 6 horas por dia) Algumas vezes por dia (em média, entre 2 e 4 horas por dia) Poucas vezes por dia (em média, entre 0 e 2 horas por dia) Não utilizo redes sociais

Prefiro não responder.

[Q13b] Se tem um smartphone, para que atividades é que o usa? *

Responda a esta pergunta apenas se as seguintes condições são verdadeiras:

A resposta for na pergunta '14 [Q13]' (Qual ou quais dos seguintes dispositivos usa? (Selecione todas as opções aplicáveis.))

 Selecione todas as opções que se apliquem Por favor, selecione **todas** as que se aplicam:

Comunicação (chamadas, mensagens de texto…) Entretenimento

Jogos

Redes sociais E-mail

Escola / trabalhos de casa

Consulta de notícias / acontecimentos da atualidade Informação de viagem / navegação

Aplicações para monitorizar a nutrição / dieta Outras aplicações de saúde (p.ex. doença crónica) Fitness / exercício físico

Saúde mental

Prefiro não responder

Outro:


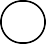


[Q13c] Se tem um smartphone, ter espaço suficiente para descarregar aplicações móveis é uma preocupação? *

Responda a esta pergunta apenas se as seguintes condições são verdadeiras:

A resposta for na pergunta '14 [Q13]' (Qual ou quais dos seguintes dispositivos usa? (Selecione todas as opções aplicáveis.))

 Escolher uma das seguintes respostas

Por favor, selecione **apenas uma** das seguintes opções:

Sim
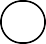
 Não
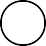
 Não sei


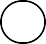
 Prefiro não responder


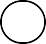


[Q14] As pessoas podem usar a internet para ver vídeos/ouvir música, jogar jogos, ir às redes

sociais, usar aplicações móveis, pesquisar algum assunto, etc. num computador ou num telemóvel ou dispositivo móvel.

Num dia normal, com que regularidade usa a internet de forma proativa (excluindo, por exemplo, ter aplicações de mensagens ligadas, mas a não serem ativamente utilizadas)? *

 Escolher uma das seguintes respostas

Por favor, selecione **apenas uma** das seguintes opções:


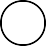
 Quase constantemente (em média, mais de 6 horas por dia) Muitas vezes por dia (em média, entre 4 e 6 horas por dia)


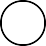
 Algumas vezes por dia (em média, entre 2 e 4 horas por dia)
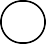
 Poucas vezes por dia (em média, entre 0 e 2 horas por dia)
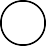
 Não utilizo a Internet num dia normal.


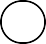
 Prefiro não responder.


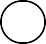


[Q15] Onde é que é mais frequente aceder à internet? *

 Escolher uma das seguintes respostas

Por favor, selecione **apenas uma** das seguintes opções:

Em casa
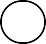
 No trabalho


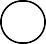
 Na universidade


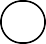
 No telemóvel (independentemente do sítio)


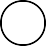
 Nas deslocações diárias (transportes públicos, trânsito, etc.)
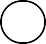
 Prefiro não responder.


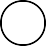
 Outro


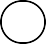


[Q16] Tem acesso consistente a Wi-Fi ou redes móveis? *

 Escolher uma das seguintes respostas

Por favor, selecione **apenas uma** das seguintes opções:

Sim
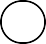
 Não
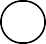
 Não sei


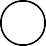
 Prefiro não responder


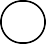


[Q17] Tem um plano de dados móveis? *

 Escolher uma das seguintes respostas

Por favor, selecione **apenas uma** das seguintes opções:

Sim
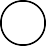
 Não Não sei

Prefiro não responder

[Q17a] Preocupa-se com o seu plano de dados móveis quando usa o seu telemóvel (por exemplo, a preocupação de que vai ficar sem dados, de que uma aplicação vai gastar muitos dados, etc.)? *

Responda a esta pergunta apenas se as seguintes condições são verdadeiras: A resposta for 'Sim' na pergunta '21 [Q17]' (Tem um plano de dados móveis?)

 Escolher uma das seguintes respostas

Por favor, selecione **apenas uma** das seguintes opções:

Sim Não

Não sei

Prefiro não responder

[Q18] Quando não tem acesso a Wi-Fi, liga os dados (3G, 4G, 5G)? *

 Escolher uma das seguintes respostas

Por favor, selecione **apenas uma** das seguintes opções:

Sim Não Não sei

Prefiro não responder

# Aplicações móveis de saúde mental

Esta secção é sobre tecnologia especificamente para a saúde mental.

[Q19]

Alguma vez usou uma aplicação móvel de saúde mental? (Selecione uma resposta.)

*Quando falamos em “aplicação móvel de saúde mental”, referimo-nos a uma aplicação no seu*

*telemóvel ou tablet que ajude a lidar com a sua saúde mental, emocional ou psicológica, ou a ter acesso a recursos de apoio à sua saúde mental, emocional ou psicológica.* *

 Escolher uma das seguintes respostas

Por favor, selecione **apenas uma** das seguintes opções:

Sim, uso atualmente uma aplicação de saúde mental

Sim, já usei uma aplicação de saúde mental, mas já não uso

Não, nunca usei uma aplicação de saúde mental, mas estou interessado em usar Não, nunca usei uma aplicação de saúde mental e não estou interessado em usar Prefiro não responder

[Q19a] O próximo conjunto de afirmações vai analisar a sua opinião sobre a utilização de aplicações móveis de saúde mental. Por favor, indique em que medida concorda com (ou discorda de) cada uma das afirmações, numa escala **de 1 (Discordo totalmente) a 5**

**(Concordo totalmente)**. *

Responda a esta pergunta apenas se as seguintes condições são verdadeiras:

-------- Scenario 1 --------

A resposta for 'Sim, uso atualmente uma aplicação de saúde mental' na pergunta '24 [Q19]' (Alguma vez usou uma aplicação móvel de saúde mental? (Selecione uma resposta.) Quando falamos em “aplicação móvel de saúde mental”, referimo-nos a uma aplicação no seu telemóvel ou tablet que ajude a lidar com a sua saúde mental, emocional ou psicológica, ou a ter acesso a recursos de apoio à sua saúde mental, emocional ou psicológica.)

-------- ou Scenario 2 --------

A resposta for 'Sim, já usei uma aplicação de saúde mental, mas já não uso' na pergunta '24 [Q19]' (Alguma vez usou uma aplicação móvel de saúde mental? (Selecione uma resposta.) Quando falamos em “aplicação móvel de saúde mental”, referimo-nos a uma aplicação no seu telemóvel ou tablet que ajude a lidar com a sua saúde mental, emocional ou psicológica, ou a ter acesso a recursos de apoio à sua saúde mental, emocional ou psicológica.)

Por favor, selecione a posição apropriada para cada elemento:

|  | **Discordo totalmente**  **(1)** | **Discordo**  **(2)** | **Não sei**  **(3)** | **Concordo**  **(4)** | **Concordo totalmente**  **(5)** | **Prefiro não responder** |
| --- | --- | --- | --- | --- | --- | --- |
| **Considero que as aplicações móveis de saúde mental podem ser úteis no meu dia a dia.** |  |  |  |  |  |  |
| **Penso que usar aplicações móveis de saúde mental aumenta as minhas hipóteses de alcançar objetivos importantes para mim.** |  |  |  |  |  |  |

|  | **Discordo totalmente**  **(1)** | **Discordo**  **(2)** | **Não sei**  **(3)** | **Concordo**  **(4)** | **Concordo totalmente**  **(5)** | **Prefiro não responder** |
| --- | --- | --- | --- | --- | --- | --- |
| **Penso que usar aplicações móveis de saúde mental me ajuda a alcançar objetivos mais rapidamente.** |  |  |  |  |  |  |
| **Penso que usar aplicações móveis de saúde mental aumenta a minha produtividade.** |  |  |  |  |  |  |

**r**

[Q19b] O próximo conjunto de afirmações vai analisar a sua opinião sobre o modo como a sua informação pessoal poderá ser usada por uma aplicação de saúde mental. Por favor, indique em que medida concorda com (ou discorda de) cada uma das afirmações, numa escala

**de 1 (Discordo totalmente) a 5 (Concordo totalmente)**. *

Responda a esta pergunta apenas se as seguintes condições são verdadeiras:

A resposta for 'Sim, uso atualmente uma aplicação de saúde mental' *ou* 'Sim, já usei uma aplicação de saúde mental, mas já não uso' na pergunta '24 [Q19]' (Alguma vez usou uma aplicação móvel de saúde mental? (Selecione uma resposta.) Quando falamos em “aplicação móvel de saúde mental”, referimo-nos a uma aplicação no seu telemóvel ou tablet que ajude a lidar com a sua saúde mental, emocional ou psicológica, ou a ter acesso a recursos de apoio à sua saúde mental, emocional ou psicológica.)

Por favor, selecione a posição apropriada para cada elemento:

|  | **Discordo totalmente**  **(1)** | **Discordo (2)** | **Não sei (3)** | **Concordo (4)** | **Concordo totalmente**  **(5)** | **Prefiro não**  **responder** |
| --- | --- | --- | --- | --- | --- | --- |
| **Sinto que o uso de aplicações móveis de saúde mental me expõe perante os outros, deixando-me desconfortável.** |  |  |  |  |  |  |
| **Acredito que o uso de aplicações móveis de saúde mental aumenta a disponibilidade de informação sobre mim que considero privada e que fica disponível para outros.** |  |  |  |  |  |  |
| **Sinto que, ao usar aplicações móveis de saúde mental, é divulgada informação sobre mim que, se usada, pode comprometer a minha privacidade.** |  |  |  |  |  |  |

|  | **Discordo totalmente**  **(1)** | **Discordo (2)** | **Não sei (3)** | **Concordo (4)** | **Concordo totalmente**  **(5)** | **Prefiro não responder** |
| --- | --- | --- | --- | --- | --- | --- |
| **Tenho receio que a minha informação pessoal colocada nas aplicações de saúde mental possa ser utilizada para outras finalidades sem que isso me seja notificado ou sem a minha autorização.** |  |  |  |  |  |  |
| **Tenho receio que as aplicações móveis de saúde mental possam partilhar informação pessoal com outros grupos sem autorização para outras finalidades.** |  |  |  |  |  |  |

**r**

### [Q19c]

O próximo conjunto de afirmações vai analisar a sua opinião sobre o modo como a sua informação pessoal poderá ser usada por uma aplicação móvel de saúde mental. Por favor,

indique em que medida concorda com (ou discorda de) cada uma das afirmações, numa escala

**de 1 (Discordo totalmente) a 5 (Concordo totalmente)**. *

Responda a esta pergunta apenas se as seguintes condições são verdadeiras:

A resposta for 'Não, nunca usei uma aplicação de saúde mental, mas estou interessado em usar' *ou* 'Não, nunca usei uma aplicação de saúde mental e não estou interessado em usar' na pergunta '24 [Q19]' (Alguma vez usou uma aplicação móvel de saúde mental? (Selecione uma resposta.) Quando falamos em “aplicação móvel de saúde mental”, referimo-nos a uma aplicação no seu telemóvel ou tablet que ajude a lidar com a sua saúde mental, emocional ou psicológica, ou a ter acesso a recursos de apoio à sua saúde mental, emocional ou psicológica.)

Por favor, selecione a posição apropriada para cada elemento:

|  | **Discordo totalmente**  **(1)** | **Discordo**  **(2)** | **Não sei**  **(3)** | **Concordo**  **(4)** | **Concordo totalmente**  **(5)** | **Prefiro não responder** |
| --- | --- | --- | --- | --- | --- | --- |
| **Sinto que, se usasse aplicações móveis de saúde mental, os outros saberiam mais sobre mim do que aquilo que me deixa confortável.** |  |  |  |  |  |  |
| **Acredito que, se usasse aplicações móveis de saúde mental, informação sobre mim que considero privada estaria mais facilmente disponível para outros do que gostaria.** |  |  |  |  |  |  |

|  | **Discordo totalmente**  **(1)** | **Discordo (2)** | **Não sei (3)** | **Concordo (4)** | **Concordo totalmente**  **(5)** | **Prefiro não responder** |
| --- | --- | --- | --- | --- | --- | --- |
| **Sinto que, se usasse aplicações móveis de saúde mental, seria divulgada informação sobre mim que, se usada, comprometeria a minha privacidade.** |  |  |  |  |  |  |
| **Tenho receio que as aplicações móveis de saúde mental possam usar a minha informação pessoal para outras finalidades sem me notificarem ou pedirem a minha autorização.** |  |  |  |  |  |  |
| **Se usasse aplicações móveis de saúde mental teria receio de que estas possam partilhar informação pessoal com outros grupos sem autorização para outras finalidades.** |  |  |  |  |  |  |

## [Q20]

O próximo conjunto de afirmações vai analisar a sua opinião sobre as aplicações móveis de saúde mental. Por favor, indique em que medida concorda com (ou discorda de) cada uma das

afirmações, numa escala **de 1 (Discordo totalmente) a 5 (Concordo totalmente)**. *

Por favor, selecione a posição apropriada para cada elemento:

|  | **Discordo totalmente**  **(1)** | **Discordo**  **(2)** | **Não sei**  **(3)** | **Concordo**  **(4)** | **Concordo totalmente**  **(5)** | **Prefiro não responder** |
| --- | --- | --- | --- | --- | --- | --- |
| **Sinto que as pessoas que são importantes para mim acham que devo usar aplicações móveis de saúde mental.** |  |  |  |  |  |  |
| **Considero que tenho o conhecimento necessário para usar aplicações móveis de saúde mental.** |  |  |  |  |  |  |
| **Considero que tenho os recursos (económicos, tecnológicos, etc.) necessários para usar aplicações móveis de saúde mental.** |  |  |  |  |  |  |
| **Considero que as aplicações móveis de saúde mental são compatíveis com outras tecnologias que uso.** |  |  |  |  |  |  |
| **Sinto mais facilidade em ter ajuda quando necessito utilizando aplicações móveis de saúde mental.** |  |  |  |  |  |  |

[Q21] Quando pensa em usar aplicações móveis de saúde mental, que aspetos são importantes para si? *

 Selecione todas as opções que se apliquem Por favor, selecione **todas** as que se aplicam:

Disponibilidade na minha língua materna Gratuitidade da aplicação

Adaptação da aplicação à minha cultura e suas especificidades A aplicação permite interação com outros

As pessoas com quem interajo na aplicação terem o mesmo contexto cultural que

eu

As pessoas com quem interajo na aplicação terem experiências de saúde mental semelhantes às minhas

Garantia de privacidade da minha informação pessoal

A aplicação não ter um efeito negativo no meu dispositivo (p. ex., não consumir demasiada bateria do telemóvel, não ocupar demasiada memória)

Existência de partes da aplicação que podem ser usadas offline

Possibilidade de a aplicação poder ser facilmente usada por pessoas com incapacidade visual

Possibilidade de a aplicação poder ser facilmente usada por pessoas com incapacidade auditiva

Prefiro não responder

Outro:

# Utilização e recursos de cuidados de saúde

Esta secção é sobre utilização e recursos de cuidados de saúde.

[Q22]

Tem atualmente um seguro de saúde (por ex. ADSE, Médis, Multicare, etc.)? *

 Escolher uma das seguintes respostas

Por favor, selecione **apenas uma** das seguintes opções:

Sim Não Não sei

Prefiro não responder

[Q22a] Sabe se o plano do seu seguro de saúde cobriria uma consulta com um profissional de saúde mental (psiquiatra, psicólogo, enfermeiro, etc.)? *

Responda a esta pergunta apenas se as seguintes condições são verdadeiras:

A resposta for 'Sim' na pergunta '30 [Q22]' (Tem atualmente um seguro de saúde (por ex. ADSE, Médis, Multicare, etc.)?)

 Escolher uma das seguintes respostas

Por favor, selecione **apenas uma** das seguintes opções:

Não, com toda a certeza.

Penso que não, mas não tenho a certeza. Não sei

Penso que sim, mas não tenho a certeza. Sim, com toda a certeza.

Prefiro não responder.

[Q23] Houve alguma altura durante os últimos 12 meses em que sentiu que poderia precisar da ajuda de um profissional devido a problemas com a sua saúde mental, emoções ou situações provocadoras de ansiedade, ou uso de álcool ou drogas / outras adições? *

 Escolher uma das seguintes respostas

Por favor, selecione **apenas uma** das seguintes opções:

Sim Não

Não sei

Prefiro não responder

### [Q23a]

Que problemas de saúde mental ou psicológica, caso existam, enfrentou nos últimos 12 meses? (Selecione todas as opções aplicáveis.) Se as opções não espelharem os seus problemas de saúde mental, sinta-se à vontade para descrever o problema nas suas próprias palavras,

selecionando a opção "Outro". *Se preferir não responder, selecione a opção “Prefiro não responder”.* *

Responda a esta pergunta apenas se as seguintes condições são verdadeiras:

A resposta for 'Sim' *ou* 'Não sei' na pergunta '32 [Q23]' (Houve alguma altura durante os últimos 12 meses em que sentiu que poderia precisar da ajuda de um profissional devido a problemas com a sua saúde mental, emoções ou situações provocadoras de ansiedade, ou uso de álcool ou drogas / outras adições?)

 Selecione todas as opções que se apliquem Por favor, selecione **todas** as que se aplicam:

Depressão Ansiedade Stress

Dificuldade em dormir Solidão

Abuso de substâncias e/ou álcool e/ou outros comportamentos aditivos Distúrbio alimentar

Obsessões e compulsões que interferem com atividades quotidianas Problemas de saúde mental após o parto

Evento de vida (p. ex., mudança, morte, doença)

Relações interpessoais (p. ex., terminei uma relação, tive uma discussão com o meu pai / a minha mãe)

Eventos psicóticos Esquizofrenia Doença bipolar

Não enfrentei qualquer problema de saúde mental ou emocional nos últimos 12 meses

Prefiro não responder Outro:

[Q24]

Nos últimos 12 meses, teve alguma consulta com o seu **médico de família ou médico de medicina geral e familiar** devido a problemas relacionados com a sua saúde mental, emoções

ou situações provocadoras de ansiedade, ou uso de álcool ou drogas/ outras adições? *

 Escolher uma das seguintes respostas

Por favor, selecione **apenas uma** das seguintes opções:

Sim Não Não sei

Prefiro não responder

[Q25]

Nos últimos 12 meses, teve alguma consulta com **qualquer outro profissional, como um**

**psiquiatra ou um psicólogo**, devido a problemas com a sua saúde mental, emoções ou situações provocadoras de ansiedade, ou uso de álcool ou drogas / outras adições? *

 Escolher uma das seguintes respostas

Por favor, selecione **apenas uma** das seguintes opções:

Sim Não Não sei

Prefiro não responder

### [Q25a]

Procurou ajuda pela sua saúde mental ou emocional ou por um problema com álcool, drogas ou outras adições? Se sim, para quais (Selecione todas as respostas aplicáveis.)? *

Responda a esta pergunta apenas se as seguintes condições são verdadeiras:

-------- Scenario 1 --------

A resposta for 'Sim' na pergunta '34 [Q24]' (Nos últimos 12 meses, teve alguma consulta com o seu médico de família ou médico de medicina geral e familiar devido a problemas relacionados com a sua saúde mental, emoções ou situações provocadoras de ansiedade, ou uso de álcool ou drogas/ outras adições?)

-------- ou Scenario 2 --------

A resposta for 'Sim' na pergunta '35 [Q25]' (Nos últimos 12 meses, teve alguma consulta com qualquer outro profissional, como um psiquiatra ou um psicólogo, devido a problemas com a sua saúde mental, emoções ou situações provocadoras de ansiedade, ou uso de álcool ou drogas / outras adições?)

 Selecione todas as opções que se apliquem Por favor, selecione **todas** as que se aplicam:

Depressão Ansiedade Stress

Dificuldade em dormir Solidão

Abuso de substâncias e/ou álcool e/ou outros comportamentos aditivos Distúrbio alimentar

Obsessões e compulsões que interferem com atividades quotidianas Problemas de saúde mental após o parto

Evento de vida (p. ex., mudança, morte, doença)

Relações interpessoais (p. ex., terminei uma relação, tive uma discussão com o meu pai / a minha mãe)

Eventos psicóticos Esquizofrenia Doença bipolar

Não enfrentei qualquer problema de saúde mental ou emocional nos últimos 12 meses.

Não sei

Prefiro não responder

Outro:

[Q25b]

Ainda está a realizar uma intervenção para estes problemas por um ou mais desses profissionais de saúde? *

Responda a esta pergunta apenas se as seguintes condições são verdadeiras:

-------- Scenario 1 --------

A resposta for 'Sim' na pergunta '34 [Q24]' (Nos últimos 12 meses, teve alguma consulta com o seu médico de família ou médico de medicina geral e familiar devido a problemas relacionados com a sua saúde mental, emoções ou situações provocadoras de ansiedade, ou uso de álcool ou drogas/ outras adições?)

-------- ou Scenario 2 --------

A resposta for 'Sim' na pergunta '35 [Q25]' (Nos últimos 12 meses, teve alguma consulta com qualquer outro profissional, como um psiquiatra ou um psicólogo, devido a problemas com a sua saúde mental, emoções ou situações provocadoras de ansiedade, ou uso de álcool ou drogas / outras adições?)

 Escolher uma das seguintes respostas

Por favor, selecione **apenas uma** das seguintes opções:

Sim Não

Não sei

Prefiro não responder

[Q26]

Nos últimos 12 meses, tentou obter ajuda de algum recurso digital, incluindo aplicações móveis ou serviços de mensagens, para problemas com a sua saúde mental, emoções, ou uso de álcool, drogas, ou outras adições? *

 Escolher uma das seguintes respostas

Por favor, selecione **apenas uma** das seguintes opções:

Sim Não

Prefiro não responder

[Q26a]

Quão útil sentiu que foi esse recurso digital? *

Responda a esta pergunta apenas se as seguintes condições são verdadeiras:

A resposta for 'Sim' na pergunta '38 [Q26]' (Nos últimos 12 meses, tentou obter ajuda de algum recurso digital, incluindo aplicações móveis ou serviços de mensagens, para problemas com a sua saúde mental, emoções, ou uso de álcool, drogas, ou outras adições?)

 Escolher uma das seguintes respostas

Por favor, selecione **apenas uma** das seguintes opções:

Bastante útil Muito útil

Moderadamente útil Pouco útil

Nada útil

Prefiro não responder

### [Q26b]

Qual foi a principal razão para não ter tentado obter ajuda de um recurso digital, incluindo aplicações móveis ou serviços de mensagens? *

Responda a esta pergunta apenas se as seguintes condições são verdadeiras:

A resposta for 'Não' na pergunta '38 [Q26]' (Nos últimos 12 meses, tentou obter ajuda de algum recurso digital, incluindo aplicações móveis ou serviços de mensagens, para problemas com a sua saúde mental, emoções, ou uso de álcool, drogas, ou outras adições?)

 Escolher uma das seguintes respostas

Por favor, selecione **apenas uma** das seguintes opções:

Fiquei melhor / deixei de precisar Queria resolver o problema por mim

Não tenho um smartphone ou computador Não conhecia estas aplicações

Não confio em aplicações móveis

Questões sobre privacidade e segurança dos dados Achei que não seria útil / não resultaria

O preço era muito elevado Não tenho tempo

Já estava a receber acompanhamento clínico tradicional / presencial Achei que não precisava

Não tenho espaço suficiente para descarregar aplicações novas Prefiro não responder

Outro

[Q27] Nos últimos 12 meses, teve contacto *online* com pessoas que têm problemas de saúde mental ou de uso de álcool, drogas, ou outras adições semelhantes aos seus por meios como as redes sociais, blogues ou fóruns online? *

 Escolher uma das seguintes respostas

Por favor, selecione **apenas uma** das seguintes opções:

Sim Não

Prefiro não responder

[Q28]

Nos últimos 12 meses, usou algum recurso digital para encontrar, contactar ou ser encaminhado para um profissional de saúde mental?

*Por exemplo, por SMS, mensagem online, chat de vídeo ou uma aplicação móvel de saúde mental ou relacionada com a saúde.* *

 Escolher uma das seguintes respostas

Por favor, selecione **apenas uma** das seguintes opções:

Sim Não

Prefiro não responder

[Q29]

Se precisasse de procurar recursos para a sua saúde mental enquanto membro da comunidade académica da Universidade do Porto, saberia onde se dirigir? *

 Escolher uma das seguintes respostas

Por favor, selecione **apenas uma** das seguintes opções:

Sim Não

Prefiro não responder

[Q30]

Utilizou algum destes recursos da Universidade do Porto? *

 Selecione todas as opções que se apliquem Por favor, selecione **todas** as que se aplicam:

Aconselhamento psicológico

SASUP – Serviço de Ação Social da Universidade do Porto

Workshops de Bem-estar e Saúde Mental (p.ex., bem-estar e saúde mental no trabalho, gestão da ansiedade, equilíbrio vida profissional e vida pessoa)

Workshops Presenciais (p.ex., gestão de tempo, mindfulness, evitar o burnout, autocuidado)

Não usei qualquer recurso da Universidade do Porto Prefiro não responder

Outro:

[Q31]

Com quem fala geralmente quando se sente triste, ansioso(a), preocupado(a) ou stressado(a)?

*

 Selecione todas as opções que se apliquem Por favor, selecione **todas** as que se aplicam:

Amigo(s) Namorado(a) Familiar(es) Professor(es) Colega(s)

Profissional(is) de saúde

SASUP – Serviços de Ação Social da Universidade do Porto Não falo com ninguém

Prefiro não responder

Outro:

## [Q32]

Qual ou quais dos seguintes recursos e estratégias é que **usa atualmente** para lidar com a sua saúde mental e/ou psicológica (por ex., quando se sente triste, ansioso(a), preocupado(a) ou

stressado(a)?) *

 Selecione todas as opções que se apliquem Por favor, selecione **todas** as que se aplicam:

Apoio informal, como falar ou passar tempo com familiares ou amigos

Apoio informal através de recurso a animais de companhia (por ex., cães, gatos, etc.)

Serviços profissionais (por ex., consultas com um psicólogo ou psiquiatra) Redes sociais (por ex., Facebook, Instagram, Twitter, Reddit)

Fóruns ou comunidades online (por ex, Mental Health Forum, BeyondBlue, SMS eSaúde)

Sites (por ex., Psic.ON – Suporte Psicológico Online da UPorto, saudemental.pt, eusinto.me)

Aplicações móveis (por ex., Headspace, 29k FJN, Hug-a-Group) Linha de Aconselhamento Psicológico do SNS24 (808 24 24 24)

Outras linhas de apoio telefónicas ou de texto (por ex., SOS Voz Amiga, Conversa Amiga)

Exercício físico Escrever

Pintar, desenhar, colorir, fotografar, etc. Fazer artesanato, costura, etc.

Ouvir música

Tocar um instrumento, cantar ou compor música Ler

Cozinhar Jogar jogos

Atualmente não uso quaisquer recursos ou estratégias para lidar com a minha saúde mental e/ou psicológica

Prefiro não responder Outro:

### [Q32a]

Por favor, indique, para cada recurso que usa atualmente, quão útil é esse recurso para a manutenção da sua saúde mental, numa escala **de 1 (Nada útil) a 5 (Extremamente útil)**. *

Responda a esta pergunta apenas se as seguintes condições são verdadeiras:

-------- Scenario 1 --------

A resposta for na pergunta '46 [Q32]' (Qual ou quais dos seguintes recursos e estratégias é que usa atualmente para lidar com a sua saúde mental e/ou psicológica (por ex., quando se sente triste, ansioso(a), preocupado(a) ou stressado(a)?))

-------- ou Scenario 2 --------

A resposta for na pergunta '46 [Q32]' (Qual ou quais dos seguintes recursos e estratégias é que usa atualmente para lidar com a sua saúde mental e/ou psicológica (por ex., quando se sente triste, ansioso(a), preocupado(a) ou stressado(a)?))

Por favor, selecione a posição apropriada para cada elemento:

|  | **Nada útil (1)** | **Pouco útil (2)** | **Razoavelmente útil (3)** | **Muito útil (4)** | **Extremamente útil (5)** | **Prefiro não responder** |
| --- | --- | --- | --- | --- | --- | --- |
| **Apoio informal, como falar ou passar tempo com familiares ou amigos** |  |  |  |  |  |  |
| **Serviços profissionais (exemplos: terapia com um psicólogo ou psiquiatra)** |  |  |  |  |  |  |
| **Redes sociais (exemplos: Facebook, Instagram, Twitter, Reddit)** |  |  |  |  |  |  |
| **Fóruns ou comunidades online (exemplos: Mental Health Forum, BeyondBlue, SMS eSaúde)** |  |  |  |  |  |  |

|  | **Nada útil**  **(1)** | **Pouco útil (2)** | **Razoavelmente útil (3)** | **Muito útil (4)** | **Extremamente útil (5)** | **Prefiro não responder** |
| --- | --- | --- | --- | --- | --- | --- |
| **Sites (exemplos: Psic.ON – Suporte Psicológico Online da UPorto, saudemental.pt, eusinto.me)** |  |  |  |  |  |  |
| **Aplicações móveis (por ex., Headspace, 29k FJN, Hug-a- Group)** |  |  |  |  |  |  |
| **Linha de Aconselhamento Psicológico do SNS24 (808 24 24 24)** |  |  |  |  |  |  |
| **Outras linhas de crise telefónicas ou de texto (p. ex., SOS Voz Amiga, Conversa Amiga)** |  |  |  |  |  |  |
| **Desporto ou atividades físicas** |  |  |  |  |  |  |
| **Escrever** |  |  |  |  |  |  |
| **Pintar, desenhar, colorir, fotografar, etc.** |  |  |  |  |  |  |
| **Fazer artesanato, costura, etc.** |  |  |  |  |  |  |

|  | **Nada útil (1)** | **Pouco útil (2)** | **Razoavelmente útil  (3)** | **Muito útil (4)** | **Extremamente útil (5)** | **Prefiro não**  **responder** |
| --- | --- | --- | --- | --- | --- | --- |
| **Ouvir música** |  |  |  |  |  |  |
| **Tocar um instrumento, cantar ou fazer música** |  |  |  |  |  |  |
| **Ler** |  |  |  |  |  |  |
| **Cozinhar** |  |  |  |  |  |  |
| **Jogar jogos** |  |  |  |  |  |  |
| **Atualmente não uso quaisquer recursos ou estratégias para gerir a minha saúde mental** |  |  |  |  |  |  |
| **Prefiro não responder** |  |  |  |  |  |  |

**r**

## [Q33]

Qual ou quais dos seguintes recursos e estratégias é que **gostaria de usar** para lidar com a sua saúde mental? *

 Selecione todas as opções que se apliquem Por favor, selecione **todas** as que se aplicam:

Apoio informal, como falar ou passar tempo com familiares ou amigos

Apoio informal através de recurso a animais de companhia (por ex., cães, gatos, etc.)

Serviços profissionais (por ex., consultas com um psicólogo ou psiquiatra) Redes sociais (por ex., Facebook, Instagram, Twitter, Reddit)

Fóruns ou comunidades online (por ex., Mental Health Forum, BeyondBlue, SMS eSaúde)

Sites (por ex., Psic.ON – Suporte Psicológico Online da UPorto, saudemental.pt, eusinto.me)

Aplicações móveis (por ex., Headspace, 29k FJN, Hug-a-Group) Linha de Aconselhamento Psicológico do SNS24 (808 24 24 24)

Outras linhas de apoio telefónicas ou de texto (por ex., SOS Voz Amiga, Conversa Amiga)

Exercício físico Escrever

Pintar, desenhar, colorir, fotografar, etc. Fazer artesanato, costura, etc.

Ouvir música

Tocar um instrumento, cantar ou compor música Ler

Cozinhar Jogar jogos

Não estou interessado(a) em usar quaisquer recursos ou estratégias para lidar com a minha saúde mental.

Prefiro não responder Outro:

### [Q33a]

Por favor, indique, para cada recurso que gostaria de usar, mas que não usa atualmente, qual a probabilidade de usar esse recurso, numa escala **de 1 (Extremamente improvável) a 5**

**(Extremamente provável)**. *

Responda a esta pergunta apenas se as seguintes condições são verdadeiras:

-------- Scenario 1 --------

A resposta for na pergunta '48 [Q33]' (Qual ou quais dos seguintes recursos e estratégias é que gostaria de usar para lidar com a sua saúde mental?)

-------- ou Scenario 2 --------

A resposta for na pergunta '48 [Q33]' (Qual ou quais dos seguintes recursos e estratégias é que gostaria de usar para lidar com a sua saúde mental?)

Por favor, selecione a posição apropriada para cada elemento:

|  | **Extremamente improvável**  **(1)** | **Improvável**  **(2)** | **Nem provável nem improvável**  **(3)** | **Provável**  **(4)** | **Extremamente provável**  **(5)** | **Prefiro não**  **responder** |
| --- | --- | --- | --- | --- | --- | --- |
| **Apoio informal, como falar ou passar tempo com familiares ou amigos** |  |  |  |  |  |  |
| **Serviços profissionais (por ex., terapia com um psicólogo ou psiquiatra)** |  |  |  |  |  |  |
| **Redes sociais (por ex., Facebook, Instagram, Twitter, Reddit)** |  |  |  |  |  |  |
| **Fóruns ou comunidades online (por ex., Mental Health Forum, BeyondBlue, SMS eSaúde)** |  |  |  |  |  |  |

|  | **Extremamente improvável**  **(1)** | **Improvável**  **(2)** | **Nem provável nem improvável**  **(3)** | **Provável**  **(4)** | **Extremamente provável**  **(5)** | **Prefiro não responder** |
| --- | --- | --- | --- | --- | --- | --- |
| **Sites (por ex., Psic.ON – Suporte Psicológico Online da UPorto, saudemental.pt, eusinto.me)** |  |  |  |  |  |  |
| **Aplicações móveis (por ex., Headspace, 29k FJN, Hug-a- Group)** |  |  |  |  |  |  |
| **Linha de Aconselhamento Psicológico do SNS24 (808 24 24 24)** |  |  |  |  |  |  |
| **Outras linhas de crise telefónicas ou de texto (por ex., SOS Voz Amiga, Conversa Amiga)** |  |  |  |  |  |  |
| **Programas de exercício ou atividades físicas** |  |  |  |  |  |  |
| **Escrever** |  |  |  |  |  |  |
| **Pintar, desenhar, colorir, fotografar, etc.** |  |  |  |  |  |  |
| **Fazer artesanato, costura, etc.** |  |  |  |  |  |  |
| **Ouvir música** |  |  |  |  |  |  |
| **Tocar um instrumento, cantar ou fazer música** |  |  |  |  |  |  |

|  | **Extremamente improvável**  **(1)** | **Improvável**  **(2)** | **Nem provável**  **nem improvável (3)** | **Provável**  **(4)** | **Extremamente provável**  **(5)** | **Prefiro não responder** |
| --- | --- | --- | --- | --- | --- | --- |
| **Ler** |  |  |  |  |  |  |
| **Cozinhar** |  |  |  |  |  |  |
| **Jogar jogos** |  |  |  |  |  |  |
| **Não estou interessado(a) em usar quaisquer recursos ou estratégias para gerir a minha saúde mental.** |  |  |  |  |  |  |
| **Prefiro não responder** |  |  |  |  |  |  |

**r**

[Q34]

Quando pensa em usar recursos digitais de saúde mental, que coisas é que gostaria de ser capaz de fazer? *

 Selecione todas as opções que se apliquem Por favor, selecione **todas** as que se aplicam:

Identificar ou reconhecer sintomas Monitorizar sintomas

Superar as emoções e pensamentos negativos Falar com outras pessoas para obter / dar apoio Ter acesso a um profissional de saúde mental

Ler sobre experiências de saúde mental de outras pessoas

Manter-me organizado e manter o controlo das tarefas e responsabilidades Expressar-me ou ter um refúgio relacionado com arte, fotografia ou escrita Distrair-me de emoções e pensamentos negativos

Obter informação sobre sintomas e condições de saúde mental

Obter informação sobre como aceder a recursos locais de saúde mental Aceder a materiais educativos sobre como lidar com o stress

Obter informação sobre como lidar com o luto ou a perda Obter informação sobre como lidar com o trauma

Obter informação sobre como lidar com problemas na relação Prefiro não responder

Outro:

## [Q35]

Com que barreiras, caso existam, se depara para aceder a recursos (p. ex., informação, serviços clínicos ou de apoio, contacto para apoio humano, etc.) relacionados com a saúde mental?

(Selecione todas as opções aplicáveis.) *

 Selecione todas as opções que se apliquem Por favor, selecione **todas** as que se aplicam:

O tempo de espera para aceder aos recursos é demasiado longo (dificuldades de acesso)

O custo para aceder aos recursos é demasiado elevado (dificuldades financeiras) Não tenho tempo

Estou preocupado(a)/tenho receios relacionados com a minha privacidade Preocupa-me que as minhas ações sejam documentadas

Tenho receio do que os outros vão pensar de mim

Não sinto que os meus problemas sejam valorizados pela minha família e tenho receio que alguém notifique os meus pais/outros familiares

As pessoas que prestam os serviços não são suficientemente sensíveis a diferenças culturais e/ou religiosas

As pessoas que prestam os serviços não são suficientemente sensíveis a diferenças de identidade sexual

Tenho dificuldades em comunicar na língua oficial do país de residência Não confio na minha capacidade de escolher a opção certa

Questiono-me se os serviços serão úteis

Tenho tido uma má experiência com estes serviços no passado O problema vai melhorar por si próprio

Acredito que os meus problemas não são suficientemente sérios para merecer estes serviços

Acho que ninguém consegue compreender os meus problemas O stress é normal na comunidade académica

Recebo muito apoio de outras fontes

Não tive quaisquer barreiras, dificuldades ou desafios Não tive qualquer necessidade de usar os recursos Prefiro não responder

Outro:

[Q36] Em que situações ou momentos é que mais precisa de apoio no que respeita à sua saúde mental? *

 Selecione todas as opções que se apliquem Por favor, selecione **todas** as que se aplicam:

Durante o dia À noite

Em casa

Na faculdade No trabalho

Antes ou depois de um exame difícil

Antes de ou durante uma situação em que necessito de interagir com outros (eventos, apresentações, etc.)

Quando tenho de lidar com um evento de vida significativo (p. ex., depois de uma separação, morte ou doença na minha família)

Prefiro não responder

Outro:

# Experiências de stress, bem-estar e saúde mental

Esta secção é sobre stress, bem-estar e saúde mental.

## [Q37]

O próximo conjunto de afirmações vai analisar a frequência com que sente stress num conjunto de situações. Por favor, indique com que frequência se sente aflito ou ansioso em cada uma das

seguintes situações, numa escala **de 1 (Nunca) a 5 (Muito frequentemente)**. *

Por favor, selecione a posição apropriada para cada elemento:

|  | **Nunca**  **(1)** | **Raramente**  **(2)** | **Às vezes (3)** | **Frequente**  **mente**  **(4)** | **Muito frequentemente**  **(5)** | **Não aplicável** | **Prefiro não**  **responder** |
| --- | --- | --- | --- | --- | --- | --- | --- |
| **Situações de problemas com relações pessoais** |  |  |  |  |  |  |  |
| **Situações de problemas familiares** |  |  |  |  |  |  |  |
| **Situações de problemas laborais** |  |  |  |  |  |  |  |
| **Situações de problemas financeiros** |  |  |  |  |  |  |  |
| **Situações de problemas académicos** |  |  |  |  |  |  |  |
| **Situações de problemas domésticos** |  |  |  |  |  |  |  |
| **Situações em que estou longe de casa** |  |  |  |  |  |  |  |
| **Eventos que não correm como planeado** |  |  |  |  |  |  |  |

## [Q38]

As questões seguintes são sobre **o mês,** **durante os últimos 12 meses,** em que se sentiu pior emocionalmente.

Durante esse mês, com que frequência é que as suas emoções interferiram, numa escala **de 1 (Nunca) a 5 (Muito frequentemente)**, com… *

Por favor, selecione a posição apropriada para cada elemento:

|  | **Nunca  (1)** | **Raramente**  **(2)** | **Às vezes (3)** | **Frequentemente (4)** | **Muito frequentemente**  **(5)** | **Não aplicável** | **Prefiro não**  **responder** |
| --- | --- | --- | --- | --- | --- | --- | --- |
| **…o seu desempenho académico?** |  |  |  |  |  |  |  |
| **…o seu desempenho profissional?** |  |  |  |  |  |  |  |
| **…as suas tarefas domésticas?** |  |  |  |  |  |  |  |
| **…a sua vida social?** |  |  |  |  |  |  |  |
| **…a sua relação com amigos e familiares?** |  |  |  |  |  |  |  |

[Q39]

As questões seguintes vão analisar o modo como se sente em relação a diferentes aspetos da sua vida.

Por favor, indique, para cada uma das seguintes questões, com que frequência se sente assim,

numa escala **de 1 (Nunca) a 5 (Muito frequentemente)**.

Com que frequência... *

Por favor, selecione a posição apropriada para cada elemento:

|  | **Nunca (1)** | **Raramente (2)** | **Às vezes (3)** | **Frequentemente (4)** | **Muito frequentemente**  **(5)** | **Não aplicável** | **Prefiro não**  **responder** |
| --- | --- | --- | --- | --- | --- | --- | --- |
| **sente falta de companhia?** |  |  |  |  |  |  |  |
| **se sente excluído?** |  |  |  |  |  |  |  |
| **se sente isolado dos outros?** |  |  |  |  |  |  |  |

## [Q40]

As questões seguintes vão analisar o modo como se sentiu durante os últimos 30 dias.

Por favor, indique, para cada uma das seguintes questões, com que frequência se sentiu assim nos últimos 30 dias, numa escala **de 1 (Nunca) a 5 (Sempre)**.

Nos últimos 30 dias, com que frequência se sentiu... *

Por favor, selecione a posição apropriada para cada elemento:

|  | **Nunca (1)** | **Raramente (2)** | **Às vezes (3)** | **Frequentemente (4)** | **Muito frequentemente**  **(5)** | **Não aplicável** | **Prefiro não responder** |
| --- | --- | --- | --- | --- | --- | --- | --- |
| **...exausto(a) sem nenhuma razão?** |  |  |  |  |  |  |  |
| **...nervoso(a)?** |  |  |  |  |  |  |  |
| **...tão nervoso(a) que nada conseguia acalmá-lo(a)?** |  |  |  |  |  |  |  |
| **...sem esperança?** |  |  |  |  |  |  |  |
| **...inquieto(a) ou agitado(a)?** |  |  |  |  |  |  |  |
| **...tão inquieto(a) que não conseguia estar parado(a)?** |  |  |  |  |  |  |  |
| **...deprimido/(a)?** |  |  |  |  |  |  |  |
| **...em constante esforço (sentiu que tudo exigia esforço)?** |  |  |  |  |  |  |  |
| **...tão triste que nada conseguia animá-lo(a)?** |  |  |  |  |  |  |  |
| **...sem valor?** |  |  |  |  |  |  |  |

# Perceções de saúde mental

Muitas pessoas sofreram (ou estão atualmente a sofrer) de uma doença mental que afeta a sua capacidade para serem funcionais em contexto familiar, relacional ou profissional. São usados muitos termos diferentes para nos referirmos a esta condição: “doença mental”, “problema de saúde mental”, “perturbação emocional”, “perturbação ou transtorno psicológico”, “desafio mental”, etc.

[Q41] Alguma vez sofreu de um problema de saúde mental? *

 Escolher uma das seguintes respostas

Por favor, selecione **apenas uma** das seguintes opções:

Sim, sofro atualmente ou já sofri Não, nunca

Prefiro não responder

### [Q41a]

Esta questão analisa o estigma associado à saúde mental. O estigma pode fazer com que as pessoas se sintam mal por algo que está fora do seu controlo. Tenha em atenção que as seguintes afirmações **não** representam necessariamente a visão da saúde mental ou da doença mental dos investigadores do projeto.

Nesta questão, será usado o termo “problema de saúde mental”. No entanto, poderão ser usados outros termos, como saúde mental, perturbação mental, condição de saúde mental, saúde psicológica, bem-estar emocional, entre outros.

Por favor, indique em que medida concorda com (ou discorda de) cada uma das seguintes afirmações, numa escala **de 1 (Discordo totalmente) a 5 (Concordo totalmente)**: *

Responda a esta pergunta apenas se as seguintes condições são verdadeiras:

A resposta for 'Sim, sofro atualmente ou já sofri' na pergunta '57 [Q41]' (Alguma vez sofreu de um problema de saúde mental?)

Por favor, selecione a posição apropriada para cada elemento:

|  | **Discordo totalmente**  **(1)** | **Discordo**  **(2)** | **Não sei**  **(3)** | **Concordo**  **(4)** | **Concordo totalmente**  **(5)** | **Prefiro e não**  **responde** |
| --- | --- | --- | --- | --- | --- | --- |
| **Penso que os estereótipos sobre pessoas com problemas de saúde mental aplicam-se a mim** |  |  |  |  |  |  |
| **Penso que, de um modo geral, consigo viver a vida como quero** |  |  |  |  |  |  |
| **Penso que os estereótipos negativos sobre as pessoas com problemas de saúde mental me fazem sentir isolado(a)** |  |  |  |  |  |  |

|  | **Discordo totalmente**  **(1)** | **Discordo (2)** | **Não sei (3)** | **Concordo (4)** | **Concordo totalmente**  **(5)** | **Prefiro não**  **responder** |
| --- | --- | --- | --- | --- | --- | --- |
| **Penso que me sinto deslocado/ excluído(a) do mundo por ter um problema de saúde mental** |  |  |  |  |  |  |
| **Penso que estar rodeado de pessoas que não têm um problema de saúde mental me faz sentir deslocado(a) ou desajustado(a)** |  |  |  |  |  |  |
| **Penso que as pessoas sem um problema de saúde mental não conseguem compreender-me.** |  |  |  |  |  |  |
| **Penso que, por ter um problema de saúde mental, ninguém teria interesse em aproximar-se de mim** |  |  |  |  |  |  |
| **Penso que por ter um problema de saúde mental não consigo contribuir para a sociedade** |  |  |  |  |  |  |
| **Penso que consigo ter uma vida plena e gratificante, apesar do meu problema de saúde mental** |  |  |  |  |  |  |

|  | **Discordo totalmente**  **(1)** | **Discordo (2)** | **Não sei**  **(3)** | **Concordo**  **(4)** | **Concordo totalmente**  **(5)** | **Prefiro não responder** |
| --- | --- | --- | --- | --- | --- | --- |
| **Penso que, na minha cultura, é aceitável receber tratamento para problemas de saúde mental** |  |  |  |  |  |  |
| **Prefiro não responder** |  |  |  |  |  |  |

## [Q42]

A questão seguinte vai analisar a sua opinião e visão das **pessoas com uma doença mental**. Nesta secção, será usado o termo “problema de saúde mental”. No entanto, poderão ser usados outros termos, como doença mental, transtorno mental, distúrbio psicológico ou condição de saúde mental.

Por favor, indique em que medida concorda com (ou discorda de) cada uma das seguintes afirmações, numa escala **de 1 (Discordo totalmente) a 5 (Concordo totalmente)**. *

Por favor, selecione a posição apropriada para cada elemento:

|  | **Discordo totalmente**  **(1)** | **Discordo (2)** | **Não sei (3)** | **Concordo (4)** | **Concordo totalmente**  **(5)** | **Prefiro não responder** |
| --- | --- | --- | --- | --- | --- | --- |
| **A maior parte das pessoas acredita que as pessoas com um problema de saúde mental poderiam superar o problema se quisessem.** |  |  |  |  |  |  |
| **A maior parte das pessoas acredita que ter uma doença mental é um sinal de fraqueza pessoal.** |  |  |  |  |  |  |
| **A maior parte das pessoas acredita que a doença mental não é uma verdadeira doença.** |  |  |  |  |  |  |
| **A maior parte das pessoas acredita que as pessoas com problemas de saúde mental são perigosas.** |  |  |  |  |  |  |

|  | **Discordo totalmente**  **(1)** | **Discordo (2)** | **Não sei (3)** | **Concordo (4)** | **Concordo totalmente**  **(5)** | **Prefiro não responder** |
| --- | --- | --- | --- | --- | --- | --- |
| **A maior parte das pessoas acredita que é melhor evitar as pessoas com uma doença mental para não ficarem mentalmente doentes também.** |  |  |  |  |  |  |
| **A maior parte das pessoas acredita que as pessoas com problemas de saúde mental são imprevisíveis.** |  |  |  |  |  |  |
| **A maior parte das pessoas não contaria a ninguém se tivesse um problema de saúde mental.** |  |  |  |  |  |  |
| **A maior parte das pessoas não contrataria alguém se soubesse que tinha uma doença mental.** |  |  |  |  |  |  |
| **Penso que consigo ter uma vida plena e gratificante, apesar do meu problema de saúde mental.** |  |  |  |  |  |  |
| **A maior parte das pessoas não votaria num político se soubesse que ele/ela tinha uma doença mental.** |  |  |  |  |  |  |

|  | **Discordo totalmente**  **(1)** | **Discordo (2)** | **Não sei (3)** | **Concordo (4)** | **Concordo totalmente**  **(5)** | **Prefiro não responder** |
| --- | --- | --- | --- | --- | --- | --- |
| **Prefiro não responder.** |  |  |  |  |  |  |

[Q43]

O que mais gostaria de nos contar? Isto inclui, mas não está limitado a, por exemplo, partilhar mais informação sobre a sua saúde mental ou bem-estar, estratégias e recursos para enfrentar o problema ou utilização de (e interesse em) tecnologia.

*Se preferir não responder, pode deixar este espaço em branco.*

Por favor, escreva aqui a sua resposta:

Caso deseje ser contactado(a) para o recebimento de uma sinopse dos resultados decorrentes [deste inquérito em estudo publicado, por favor preencha este formulário (https://inqueritos.up.pt/index.php?r=survey/index&sid=489829&lang=pt)](https://inqueritos.up.pt/index.php?r=survey/index&sid=489829&lang=pt).

Obrigado pela sua participação e contribuição para a investigação!

Obrigado por ter concluído este inquérito.

Survey - Academic Community

**Survey on How Mobile Technologies Can Fulfill the Unmet Mental Health and Well-being Needs of the Academic Community of the University of Porto**

The purpose of this survey is to understand the unmet mental health needs of the academic community (i.e., students, faculty, and staff) and the strategies they currently use to address their mental health, with in order to provide adequate resources (including digital tools). Previous research has revealed that university students encounter mental health problems and lack of wellbeing that are often not identified and treated, and that better access to targeted mental health resources is needed. However, there is a lack of research on the mental health needs of university students and the availability and use of technology to meet those needs. Furthermore, there is no study that addresses, in addition to the unmet needs of students, those of teachers and staff. Although there is a consensus in the literature that adolescents and young adults are at greater risk of psychological and emotional disorders, a complete picture of the academic community cannot be constructed without these other two groups. Such an inclusion also has the advantage of generating an understanding that allows for an approximation to the reality of the Portuguese population, paying special attention to potential biases.

We intend to investigate the following questions:

1. What are the current unmet mental health and well-being needs of the academic community?
2. What are the current tools and strategies used to address health and well-being?
3. How can digital tools, such as mobile apps, fulfill unmet needs?
4. What factors might influence the adoption/development of mental health apps?

This survey is being conducted as part of a PhD thesis for the PhD Programme in Health Data Science at the FMUP. The person responsible for collecting data is the student Diogo Nogueira Leite (202002508), who can be contacted at up202002508@up.pt for further questions.

This survey is comprised of 60 question(s).

Demographic and basic information

This part of the survey is about your basic and demographic information. This data is exclusively used for the characterisation of the sample of answers and will not be used individually or for purposes other than global characterisation.

[Q1] How old are you? (Please only enter the number of years old you are. If you do not wish to answer, please enter 0) *

Please write your answer here:

[Q2]

What gender do you identify as? *

Please select **only one** of the following options:

Male Female

Transgender man

Transgender woman

Genderqueer/ Non-conforming / Non-binary Uncertain about gender identity I prefer not to answer.

Other

[Q3]

Select the option that best describes your situation. (Select all that apply.) *

Please select **all** that apply:

Full-time student in the first cycle of studies (Bachelor’s Degree) Full-time student in the second cycle of studies (Master’s Degree)

Full-time student in post-graduate studies/specialisation Full-time student in the third cycle of studies (Doctoral Degree) Part-time student in the first cycle of studies

Part-time student in the second cycle of studies

Part-time student in post-graduate studies/specialisation Part-time student in the third cycle of studies

Professor (regardless of the professional category) Staff of the University of Porto and/or its Organic Units I prefer not to answer

Other:

[Q4] Please select the option that best describes your occupational status. *

Please select **only one** of the following options:

Full-time employee Part-time employee

Full-time student (includes scholarship holders)

Student worker

Unemployed, looking for a job

Unemployed, not looking for a job I prefer not to answer

Other

[Q5]

When do you attend most of your classes at the University of Porto? (If you are not a student or you are, e.g., working on a thesis without a curricular component, please select "Not applicable") *

Please select **only one** of the following options:

Daytime

Evening Not applicable

I prefer not to answer Other

[Q6]

What is your ethnicity? (Please select one answer. If you have multiple ethnicities, select the 'more than one ethnicity' option. Categorisation based on Census 2021) * *

Please select **only one** of the following options:

White/White Portuguese/of European origin

Black/Black Portuguese/Afro-descendant/of African origin. Asian/Portuguese of Asian origin/of Asian origin

Gypsy/Portuguese Gypsy/Roma More than one ethnicity

I prefer not to answer

Other

[Q7]

Please select the option that best describes your current marital status. *

Please select **only one** of the following options:

Single

In a committed relationship, but not married Married

Widower

Divorced Separated

I prefer not to answer

Other

[Q8]

Do you have children or dependents? *

Please select **only one** of the following options:

Yes No

I prefer not to answer

[Q9]

What is your current housing situation? (Select one answer - if in doubt, consider the situation you have lived in the longest) * *

Please select **only one** of the following options:

I live alone

I live with my partner or spouse I live with roommates

I live with my family I prefer not to answer

Other

[Q10]

Are you currently homeless? *

Please select **only one** of the following options:

Yes No

I prefer not to answer

[Q11]

What is the current annual income of your household?

*

Please select **only one** of the following options:

Less than €5,000 (ca. €416/month) €5,001-10,000 (€417-833/month)

€10,001 – 13,500 (€834-1,125/month)

€13,501 – 19,000 (€1,126-1,583/month)

€19,001 – 27,500 (€1,584-2,291/month)

€27,501 – 32,500 (€2,292-2,708/month)

€32,501 – 40,000 (€2,709-3,333/month)

€40,001 – 50,000 (€3,334-4,166/month)

€50,001 – 100,000 (€4,167-8,333/month)

€100,001 – 250,000 (€8,334-20,833/month)

More than €250,000 (>€20,833/month) I don’t know

I prefer not to answer

[Q12]

Do you have a disability?

For the purposes of this survey, disability is defined as a mental or physical health condition that has lasted for more than 6 months and which limits major life activities, but which is not the result of a serious mental illness… *

Please select **only one** of the following options:

Yes No

I prefer not to answer

[Q12a]

What type of disability do you have? (Please select all that apply.)

For the purposes of this survey, disability is defined as a mental or physical health condition that has lasted for more than 6 months and which limits major life activities, but which is not the result of a serious mental illness… *

Answer this question only if the following conditions are true:

The answer is 'Yes' in question '12 [Q12]' (Do you have a disability? For the purposes of this survey, disability is defined as a mental or physical health condition that has lasted for more than 6 months and which limits major life activities, but which is not the result of a serious mental illness.)

Please select **only one** of the following options:

Mild to moderate mental disability Physical/Mobility disability

Chronic health problem (including chronic pain, cancer pathology, neurological pathology)

Visual impairment Hearing impairment I prefer not to answer

Other

# Use of technology in general and of smartphone

This section is about the use of technology and smartphone.

[Q13] Which of the following devices do you use?

(Select all that apply.) *

 Select all that apply

Please select **all** that apply:

Computer (laptop or desktop) Smartphone

Tablet

Mobile phone, but not a smartphone

Wearables (smartwatches, activity bracelets, etc.) I don’t use any of the previous options

I prefer not to answer

### [Q13a]

On a regular day, how often do you use a computer or mobile device to access social media?

*Social media can include Facebook, Instagram, Twitter, Snapchat, LinkedIn, TikTok, Pinterest, Telegram, WeChat, Weibo, etc. **

Answer this question only if the following conditions are true:

-------- Scenario 1 --------

The answer is in question '14 [Q13]' (Which of the following device(s) do you use? (Select all that apply.))

-------- or Scenario 2 --------

The answer is in question '14 [Q13]' (Which of the following device(s) do you use? (Select all that apply.))

-------- or Scenario 3 --------

The answer is in question '14 [Q13]' (Which of the following device(s) do you use? (Select all that apply.))

 Select one of the following answers

Please select **only one** of the following options:

Almost constantly (on average more than 6 hours a day) Many times a day (on average between 4 and 6 hours a day) Occasionally (on average between 2 and 4 hours a day) A few times a day (on average between 0 and 2 hours a day)

I don’t use social media

I prefer not to answer

[Q13b] If you have a smartphone, what activities do you use it for? *

Answer this question only if the following conditions are true:

The answer is in question '14 [Q13]' (Which of the following device(s) do you use? (Select all that apply.))

 Select all that apply

Please select **all** that apply:

Communication (calls, text messages...)

Entertainment

Games

Social media E-mail

School / homework

News / current events Travel information / navigation

Apps to track nutrition / diet Other health apps (e.g., chronic illness) Fitness / exercise

Mental health

I prefer not to answer

Other:

[Q13c] If you have a smartphone, is having enough space to download apps a concern? *

Answer this question only if the following conditions are true:

The answer is in question '14 [Q13]' (Which of the following device(s) do you use? (Select all that apply.))

 Select one of the following answers

Please select **only one** of the following options:

Yes. No I don’t know

I prefer not to answer.

[Q14] People can use the internet to watch videos/listen to music, play games, go on social media, use apps, research something, etc. on a computer or mobile phone or device.

On a regular day, how regularly do you proactively use the internet (excluding, for example, having messaging apps turned on but not being actively used)? *

 Select one of the following answers

Please select **only one** of the following options:

Almost constantly (on average more than 6 hours a day) Many times a day (on average between 4 and 6 hours a day)

Occasionally (on average between 2 and 4 hours a day) A few times a day (on average between 2 and 4 hours a day) I don’t use the Internet on a regular day

I prefer not to answer

[Q15] Where do you most often access the Internet? *

 Select one of the following answers

Please select **only one** of the following options:

At home At work

At university

On your mobile phone (wherever you are)

In daily commuting (public transport, traffic, etc.) I prefer not to answer

Other

[Q16] Do you have consistent access to Wi-Fi or mobile networks? *

 Select one of the following answers

Please select **only one** of the following options:

Yes No I don’t know

I prefer not to answer

[Q17] Do you have a mobile data plan? *

 Select one of the following answers

Please select **only one** of the following options:

Yes No I don’t know

I prefer not to answer

[Q17a] Do you worry about your mobile data plan when you use your phone (e.g., worry that you will run out of data, that an app will use up too much data, etc.)? *

Answer this question only if the following conditions are true:

The answer is 'Yes' to question '21 [Q17]' (Do you have a mobile data plan?)

 Select one of the following answers

Please select **only one** of the following options:

Yes No I don’t know

I prefer not to answer

[Q18] When you don't have access to Wi-Fi, do you switch on data (3G, 4G, 5G)? *

 Select one of the following answers

Please select **only one** of the following options:

Yes No I don’t know

I prefer not to answer

Mental health mobile apps

This section is about technology specifically designed for mental health.

[Q19]

Have you ever used a mental health app? (Select one answer.)

*When we say, 'mental health app', we mean an app on your mobile phone or tablet that helps you to manage your mental, emotional, or psychological health, or to access resources to support your mental, emotional, or psychological health.* *

 Select one of the following answers

Please select **only one** of the following options:

Yes, I am currently using a mental health app

Yes, I have used a mental health app, but I don't use it anymore

No, I have never used a mental health app but am interested in using one No, I have never used a mental health app and I am not interested in using one I prefer not to answer

[Q19a] The next set of statements will look at your views on the use of mental health apps. Please indicate the extent to which you agree (or disagree) with each of the statements, on a scale of **1 (Strongly disagree) to 5 (Strongly agree).** *

Answer this question only if the following conditions are true:

-------- Scenario 1 --------

The answer is 'Yes, I currently use a mental health app' in question '24 [Q19]' (Have you ever used a mental health app? (Select one answer.) When we say, 'mental health app', we mean an app on your mobile phone or tablet that helps you to manage your mental, emotional, or psychological health, or to access resources to support your mental, emotional, or psychological health).

-------- or Scenario 2 --------

The answer is 'Yes, I have used a mental health app, but I don’t use it anymore’ in question '24 [Q19]' (Have you ever used a mental health app? (Select one answer.) When we say, 'mental health app', we mean an app on your mobile phone or tablet that helps you to manage your mental, emotional, or psychological health, or to access resources to support your mental, emotional, or psychological health).

Please select the appropriate position for each element:

|  | **Strongly disagree**  **(1)** | **Disagree**  **(2)** | **I don’t know**  **(3)** | **Agree**  **(4)** | **Strongly agree**  **(5)** | **I prefer not**  **to answer** |
| --- | --- | --- | --- | --- | --- | --- |
| **I consider that mental health apps can be useful in my daily life.** |  |  |  |  |  |  |
| **I think using mental health apps increases my chances of achieving goals that are important to me.** |  |  |  |  |  |  |

|  | **Strongly disagree**  **(1)** | **Disagree**  **(2)** | **I don’t know**  **(3)** | **Agree**  **(4)** | **Strongly agree**  **(5)** | **I prefer not**  **to answer** |
| --- | --- | --- | --- | --- | --- | --- |
| **I think using mental health apps helps me achieve my goals faster.** |  |  |  |  |  |  |
| **I think using mental health apps increases my productivity.** |  |  |  |  |  |  |

[Q19b] The next set of statements will look at your views on how your personal data may be used by a mental health app. Please indicate the extent to which you agree (or disagree) with each of the statements, on a scale from **1 (Strongly disagree) to 5 (Strongly agree).** *

Only answer this question if the following conditions are true:

The answer is 'Yes, I currently use a mental health app' or 'Yes, I have used a mental health app, but I don’t use it anymore’ in question '24 [Q19]' (Have you ever used a mental health mobile app? (Select one answer.) When we say, 'mental health app', we mean an app on your mobile phone or tablet that helps you to manage your mental, emotional, or psychological health, or to access resources to support your mental, emotional, or psychological health).

Please select the appropriate position for each element:

|  | **Strongly disagree**  **(1)** | **Disagree**  **(2)** | **I don’t know**  **(3)** | **Agree**  **(4)** | **Strongly agree**  **(5)** | **I prefer not**  **to answer** |
| --- | --- | --- | --- | --- | --- | --- |
|  |  |  |  |  |  |  |
|  |  |  |  |  |  |  |
|  |  |  |  |  |  |  |

|  | **Strongly disagree**  **(1)** | **Disagree**  **(2)** | **I don’t know**  **(3)** | **Agree**  **(4)** | **Strongly agree**  **(5)** | **I prefer not**  **to answer** |
| --- | --- | --- | --- | --- | --- | --- |
| **I am concerned that my personal data placed on mental health apps may be used for other purposes without notifying me or asking for my permission.** |  |  |  |  |  |  |
| **When I give personal information to use mental health apps, I am concerned that it may use my information for other purposes.** |  |  |  |  |  |  |
| **I am concerned that mental health apps may share personal information with other groups without my permission for other purposes.** |  |  |  |  |  |  |

### [Q19c]

The next set of statements will look at your views on how your personal data may be used by a mental health app. Please indicate the extent to which you agree (or disagree) with each of the statements, on a scale from **1 (Strongly disagree) to 5 (Strongly agree).** *

Only answer this question if the following conditions are true:

The answer is 'No, I have never used a mental health app, but I am interested in using it' or 'No, I have never used a mental health app and I am not interested in using it' in question '24 [Q19]' (Have you ever used a mental health mobile app? (Select one answer.) When we say, 'mental health app', we mean an app on your mobile phone or tablet that helps you to manage your mental, emotional, or psychological health, or to access resources to support your mental, emotional, or psychological health).

Please select the appropriate position for each element:

|  |  |  |  |  |  |  |
| --- | --- | --- | --- | --- | --- | --- |
|  |  |  |  |  |  |  |
|  |  |  |  |  |  |  |

|  |  |  |  |  |  |  |
| --- | --- | --- | --- | --- | --- | --- |

## [Q20]

The next set of statements will look at your views on mental health apps. Please indicate the extent to which you agree (or disagree) with each of the statements, on a scale of **1 (Strongly disagree) to 5 (Strongly agree).** *

Please select the appropriate position for each element:

# Healthcare use and resources

This section is about healthcare use and resources.

[Q22]

Do you currently have a health insurance (e.g., ADSE, Médis, Multicare, etc.)? *

 Select one of the following answers

Please select **only one** of the following options:

Yes No I don’t know

I prefer not to answer

[Q22a] Do you know if your health insurance plan would cover an appointment with a mental health professional (psychiatrist, psychologist, nurse, etc.)? *

Answer this question only if the following conditions are true:

If the answer is 'Yes' to question '30 [Q22]' (Do you currently have a health insurance (e.g., ADSE, Médis, Multicare, etc.)?

 Select one of the following answers

Please select **only one** of the following options:

No, for sure

I don't think so, but I'm not sure I don’t know

I think so, but I’m not sure Yes, sure

I prefer not to answer

[Q23] Has there ever been a time during the last 12 months when you felt you might need help from a professional because of problems with your mental health, anxiety-provoking emotions or situations, or alcohol or drug use / other addictions? *

 Select one of the following answers

Please select **only one** of the following options:

Yes No I don’t know

I prefer not to answer

### [Q23a]

What mental or psychological health conditions, if any, have you faced in the last 12 months? (Select all that apply.) If the options do not mirror your mental health problems, feel free to describe the problem in your own words, by selecting the option "Other". *If you prefer not to answer, select the "I prefer not to answer" option.* *

Only answer this question if the following conditions are true:

The answer is 'Yes' or 'I don't know' in question '32 [Q23]' (Has there ever been a time during the last 12 months when you felt you might need help from a professional because of problems with your mental health, anxiety-provoking emotions or situations, or alcohol or drug use / other addictions?)

 Select all that apply. Please select **all** that apply:

Depression Anxiety Stress

Difficulty in sleeping Loneliness

Substance and/or alcohol abuse and/or other addictive behaviours Eating disorder

Obsessions and compulsions that interfere with daily activities Mental health problems after childbirth

Life event (e.g., change, death, illness)

Interpersonal relationships (e.g., ended a relationship, had an argument with my father/mother)

Psychotic events Schizophrenia Bipolar disorder

I have not faced any mental or emotional health problems in the last 12 months

I prefer not to answer Other:

[Q24]

In the last 12 months, have you had any appointments with your **GP or general practitioner** about problems related to your mental health, emotions or anxiety-provoking emotions or situations, or alcohol or drug use/other addictions? *

 Select one of the following answers

Please select **only one** of the following options:

Yes No I don’t know

I prefer not to answer

[Q25]

In the last 12 months, have you had any appointment with **any other professional such as a psychiatrist or a psychologist**, due to problems with your mental health, anxiety-provoking emotions or situations, or alcohol or drug use/other addictions? *

 Select one of the following answers

Please select **only one** of the following options:

Yes No I don’t know

I prefer not to answer

### [Q25a]

Have you sought help for your mental or emotional health or a problem with alcohol, drugs, or other addictions? If yes, for which (Select all that apply.)? *

Answer this question only if the following conditions are true:

-------- Scenario 1 --------

The answer is 'Yes' to question '34 [Q24]' (In the last 12 months, have you had an appointment with your GP or general practitioner about problems related to your mental health, anxiety-provoking emotions or situations, or alcohol or drug use/other addictions?)

-------- or Scenario 2 --------

The answer is 'Yes' to question '35 [Q25]' (In the last 12 months, have you had any appointment with any other professional such as a psychiatrist or a psychologist, due to problems with your mental health, anxiety-provoking emotions or situations, or alcohol or drug use/other addictions?)

 Select all the options that apply Please select **all** that apply:

Depression Anxiety Stress

Difficulty in sleeping Loneliness

Substance and/or alcohol abuse and/or other addictive behaviours Eating disorder

Obsessions and compulsions that interfere with daily activities Mental health problems after childbirth

Life event (e.g., change, death, illness)

Interpersonal relationships (e.g., ended a relationship, had an argument with my father/mother)

Psychotic events Schizophrenia Bipolar disorder

I have not faced any mental or emotional health problems in the last 12 months

I don’t know. I prefer not to answer

Other:

[Q25b]

Are you still having an intervention for these problems by one or more of these health professionals? *

Answer this question only if the following conditions are true:

-------- Scenario 1 --------

The answer is 'Yes' to question '34 [Q24]' (In the last 12 months, have you had an appointment with your GP or general practitioner about problems related to your mental health, anxiety-provoking emotions or situations, or alcohol or drug use/other addictions?)

-------- or Scenario 2 --------

The answer is 'Yes' to question '35 [Q25]' (In the last 12 months, have you had any appointment with any other professional such as a psychiatrist or a psychologist, due to problems with your mental health, anxiety-provoking emotions or situations, or alcohol or drug use/other addictions?)

 Select one of the following answers

Please select **only one** of the following options:

Yes No I don’t know

I prefer not to answer

[Q26]

In the last 12 months, have you tried to get help from any digital resource, including apps or messaging services, for problems with your mental health, emotions, or use of alcohol, drugs, or other addictions? *

 Select one of the following answers

Please select **only one** of the following options:

Yes No

I prefer not to answer

[Q26a]

How useful did you feel this digital resource was? *

Only answer this question if the following conditions are true:

The answer is 'Yes' to question '38 [Q26]' (In the last 12 months, have you tried to get help from any digital resource, including apps or messaging services, for problems with your mental health, emotions, or use of alcohol, drugs, or other addictions?)

 Select one of the following answers

Please select **only one** of the following options:

Very useful Quite useful

Reasonably useful Somewhat useful

Not useful

I prefer not to answer

### [Q26b]

What was the main reason you did not try to get help from a digital resource, including apps or messaging services? *

Only answer this question if the following conditions are true:

The answer is 'No' in question '38 [Q26]' (In the last 12 months, have you tried to get help from any digital resource, including apps or messaging services, for problems with your mental health, emotions, or use of alcohol, drugs, or other addictions?)

 Select one of the following answers

Please select **only one** of the following options:

I felt better / no longer needed I wanted to solve the problem by myself

I don't own a smartphone or a computer I did not know these apps

I don’t trust apps

Privacy and data security issues I didn't think it would be useful / wouldn't work

It was very expensive I don’t have time for it

I was already receiving traditional / face-to-face clinical follow-up I thought it was not necessary

I don't have enough space to download new apps I prefer not to answer

Other

[Q27] In the last 12 months, have you had online contact with people who have mental health problems or use alcohol, drugs, or other addictions similar to yours through means such as social media, blogs, or online forums? *

 Select one of the following answers

Please select **only one** of the following options:

Yes No

I prefer not to answer

[Q28]

In the last 12 months, have you used any digital resources to find, contact or be referred to a mental health professional?

*For example, by SMS, online message, video chat or a mental health or health-related app*.* *

 Select one of the following answers

Please select **only one** of the following options:

Yes No

I prefer not to answer

[Q29]

If you needed to seek out resources for your mental health as a member of the academic community at the University of Porto, would you know where to go*? *

 Select one of the following answers

Please select **only one** of the following options:

Yes No

I prefer not to answer

[Q30]

Have you used any of these resources from the University of Porto? *

 Select all that apply Please select **all** that apply:

Psychological counselling

SASUP – *Serviços de Ação Social da Universidade do Porto*

Wellness and Mental Health Workshops (e.g., wellness and mental health at work, anxiety management, work-life balance)

Face-to-Face Workshops (e.g., time management, mindfulness, avoiding burnout, self-care)

I did not use any resources from the University of Porto I prefer not to answer

Other:

[Q31]

Who do you usually talk to when you feel sad, anxious, worried, or stressed? *

 Select all the options that apply Please select **all** that apply:

Friend(s)

Partner

Family member(s) Professor(s)

Coworker(s)

Health professional(s)

SASUP – *Serviços de Ação Social da Universidade do Porto* I don't talk to anyone

I prefer not to answer

Other:

## [Q32]

Which of the following resources and strategies do you currently use to cope with your mental and/or psychological health (e.g., when you feel sad, anxious, worried, or stressed? *

 Select all that apply Please select **all** that apply:

Informal support, such as talking or spending time with family or friends

Informal support through the use of pets (e.g., dogs, cats, etc.)

Professional services (e.g., appointment with a psychologist or psychiatrist) Social media (e.g., Facebook, Instagram, Twitter, Reddit)

Online forums or communities (e.g., Mental Health Forum, BeyondBlue, SMS eHealth)

Websites (e.g., Psic.ON - Online Psychological Support from UPorto, saudemental.pt, eusinto.me)

Apps (e.g., Headspace, 29k FJN, Hug-a-Group)

SNS24 Psychological Counselling Line (808 24 24 24). Other telephone or text helplines (e.g., SOS Voz Amiga, Conversa Amiga)

Physical exercise Writing

Painting, drawing, colouring, photographing, etc. Doing handicrafts, sewing, etc.

Listening to music

Playing an instrument, singing, or composing Reading

Cooking Playing games

I currently do not use any resources or strategies to deal with my mental and/or psychological health

I prefer not to answer Other:

### [Q32a]

Please indicate, for each resource you currently use, how useful that resource is for maintaining your mental health, on a scale of **1 (Not at all useful) to 5 (Extremely useful)**. *

Answer this question only if the following conditions are true:

-------- Scenario 1 --------

The answer is in question '46 [Q32]' (Which of the following resources and strategies do you currently use to manage your mental and/or psychological health (e.g., when you feel sad, anxious, worried, or stressed)?

-------- or Scenario 2 --------

The answer is in question '46 [Q32]' (Which of the following resources and strategies do you currently use to manage your mental and/or psychological health (e.g., when you feel sad, anxious, worried, or stressed?))

Please select the appropriate position for each element:

|  | **Not at all useful (1)** | **Somewhat useful (2)** | **Reasonably useful (3)** | **Very useful (4)** | **Extremely useful (5)** | **I prefer not to answer** |
| --- | --- | --- | --- | --- | --- | --- |
| **Informal support, such as talking or spending time with family or friends** |  |  |  |  |  |  |
| **Professional services (examples: therapy with a psychologist or psychiatrist)** |  |  |  |  |  |  |
| **Social media (e.g., Facebook, Instagram, Twitter, Reddit)** |  |  |  |  |  |  |
| **Online forums or communities (e.g., Mental Health Forum, BeyondBlue, SMS eHealth)** |  |  |  |  |  |  |

|  | **Not at all useful (1)** | **Somewhat useful (2)** | **Reasonably useful (3)** | **Very useful (4)** | **Extremely useful (5)** | **I prefer not to answer** |
| --- | --- | --- | --- | --- | --- | --- |
| **Websites (e.g., Psic.ON - Online Psychological Support from UPorto, saudemental.pt, eusinto.me)** |  |  |  |  |  |  |
| **Apps (e.g., Headspace, 29k FJN, Hug-a-Group)** |  |  |  |  |  |  |
| **SNS24 Psychological Counselling Line (808 24 24 24)** |  |  |  |  |  |  |
| **Other telephone or text crisis helplines (e.g., SOS Friend Voice, Friend Talk)** |  |  |  |  |  |  |
| **Sports or physical activities** |  |  |  |  |  |  |
| **Writing** |  |  |  |  |  |  |
| **Painting, drawing, colouring, photographing, etc.** |  |  |  |  |  |  |
| **Doing handicrafts, sewing, etc.** |  |  |  |  |  |  |

|  | **Not at all useful (1)** | **Somewhat useful (2)** | **Reasonably useful (3)** | **Very useful (4)** | **Extremely useful (5)** | **I prefer not to answer** |
| --- | --- | --- | --- | --- | --- | --- |
| **Listening to music** |  |  |  |  |  |  |
| **Playing an instrument, singing, or composing** |  |  |  |  |  |  |
| **Reading** |  |  |  |  |  |  |
| **Cooking** |  |  |  |  |  |  |
| **Playing games** |  |  |  |  |  |  |
| **I do not currently use any resources or strategies to manage my mental health** |  |  |  |  |  |  |
| **I prefer not to answer** |  |  |  |  |  |  |

## [Q33]

Which of the following resources and strategies **would you like** to use to address your mental health? *

 Select all that apply Please select **all** that apply:

Informal support, such as talking or spending time with family or friends

Informal support through the use of pets (e.g., dogs, cats, etc.)

Professional services (e.g., appointments with a psychologist or psychiatrist)

Social media (e.g., Facebook, Instagram, Twitter, Reddit)

Online forums or communities (e.g., Mental Health Forum, BeyondBlue, SMS eHealth)

Websites (e.g., Psic.ON - Online Psychological Support from UPorto, saudemental.pt, eusinto.me)

Apps (e.g., Headspace, 29k FJN, Hug-a-Group)

SNS24 Psychological Counselling Line (808 24 24 24)

Other telephone or text helplines (e.g. SOS Voz Amiga, Conversa Amiga)

Physical exercise Writing

Painting, drawing, colouring, photographing, etc. Doing handicrafts, sewing, etc.

Listening to music

Playing an instrument, singing, or composing Reading

Cooking Playing games

I currently do not use any resources or strategies to deal with my mental and/or psychological health

I prefer not to answer Other:

### [Q33a]

Please indicate, for each resource you would like to use but do not currently use, how likely you are to use that resource, on a scale of **1 (Extremely unlikely) to 5 (Extremely likely).** *

Answer this question only if the following conditions are true:

-------- Scenario 1 --------

The answer is in question '48 [Q33]' (Which of the following resources and strategies would you like to use to manage your mental health?)

-------- or Scenario 2 --------

The answer is in question '48 [Q33]' (Which of the following resources and strategies would you like to use to manage your mental health?)

Please select the appropriate position for each element:

|  | **Extremely unlikely**  **(1)** | **Unlikely**  **(2)** | **Neither likely**  **nor unlikely**  **(3)** | **Likely**  **(4)** | **Extremely**  **likely**  **(5)** | **I prefer not to**  **answer** |
| --- | --- | --- | --- | --- | --- | --- |
| **Informal support, such as talking or spending time with family or friends** |  |  |  |  |  |  |
| **Professional services (e.g., therapy with a psychologist or psychiatrist)** |  |  |  |  |  |  |
| **Social media (e.g., Facebook, Instagram, Twitter, Reddit)** |  |  |  |  |  |  |
| **Online forums or communities (e.g., Mental Health Forum, BeyondBlue, SMS eHealth)** |  |  |  |  |  |  |

|  | **Extremely unlikely**  **(1)** | **Unlikely**  **(2)** | **Neither likely**  **nor unlikely**  **(3)** | **Likely**  **(4)** | **Extremely**  **likely**  **(5)** | **I prefer not to**  **answer** |
| --- | --- | --- | --- | --- | --- | --- |
| **Websites (e.g., Psic.ON - Online Psychological Support from UPorto, saudemental.pt, eusinto.me)** |  |  |  |  |  |  |
| **Apps (e.g., Headspace, 29k FJN, Hug-a-Group)** |  |  |  |  |  |  |
| **SNS24 Psychological Counselling Line (808 24 24 24)** |  |  |  |  |  |  |
| **Other telephone or text crisis lines (e.g., SOS Friend Voice, Friend Talk)** |  |  |  |  |  |  |
| **Exercise or physical activities** |  |  |  |  |  |  |
| **Writing** |  |  |  |  |  |  |
| **Painting, drawing, colouring, photographing, etc.** |  |  |  |  |  |  |
| **Doing handicrafts, sewing, etc.** |  |  |  |  |  |  |
| **Listening to music** |  |  |  |  |  |  |
| **Playing an instrument, singing, or composing** |  |  |  |  |  |  |

|  | **Extremely unlikely**  **(1)** | **Unlikely**  **(2)** | **Neither likely**  **nor unlikely**  **(3)** | **Likely**  **(4)** | **Extremely**  **likely**  **(5)** | **I prefer not to**  **answer** |
| --- | --- | --- | --- | --- | --- | --- |
| **Reading** |  |  |  |  |  |  |
| **Cooking** |  |  |  |  |  |  |
| **Playing games** |  |  |  |  |  |  |
| **I am not interested in using any resources or strategies to manage my mental health.** |  |  |  |  |  |  |
| **I prefer not to answer** |  |  |  |  |  |  |

[Q34]

When you think about using digital mental health resources, what things would you like to be able to do? *

 Select all that apply Please select **all** that apply:

Identify or recognise symptoms Monitoring symptoms

Overcoming negative emotions and thoughts Talking to others for support Access to a mental health professional

Read about other people's experiences of mental health

Keep myself organized, and keep track of tasks and responsibilities Express myself or have a refuge related to art, photography or writing Distract myself from negative emotions and thoughts

Obtaining information on symptoms and mental health conditions

Obtain information on how to access local mental health resources Access educational materials on how to deal with stress

Obtaining information on how to deal with grief or loss Obtaining information on how to deal with trauma

Get information on how to deal with problems in the relationship. I prefer not to answer

Other:

## [Q35]

What challenges, if any, do you face in accessing resources (e.g., information, clinical or support services, contact for human support, etc.) related to mental health?

(Select all that apply.) *

 Select all that apply Please select **all** that apply:

The waiting time to access resources is too long (access difficulties).

The cost of accessing resources is too high (financial difficulties) I don’t have time

I am worried/I worry about my privacy

I am concerned that my actions are documented

I am afraid of what others will think of me

I do not feel that my problems are valued by my family, and I am afraid that someone will notify my parents/other relatives

Service providers are not sufficiently sensitive to cultural and/or religious differences

Service providers are not sufficiently sensitive to differences in sexual identity

I have difficulties communicating in the official language of the country of residence I don't trust my ability to choose the right option

I wonder if the services are useful

I have had a bad experience with these services in the past The problem will improve on its own

I believe my problems are not serious enough to merit these services

I don't think anyone can understand my problems Stress is normal in the academic community

I get a lot of support from other sources

I faced no obstacles, difficulties or challenges I had no need to use the resources I prefer not to answer

Other:

[Q36] In what situations or moments do you most need support regarding your mental health? *

 Select all the options that apply Please select **all** that apply:

During the day At night

At home

At college At work

Before or after a difficult exam

Before or during a situation where I need to interact with others (events, presentations, etc.)

When I have to deal with a significant life event (e.g., after a separation, death, or illness in my family)

I prefer not to answer

Other:

# Experiences of stress, well-being, and mental health

This section is about stress, wellbeing, and mental health.

## [Q37]

The next set of statements will look at how often you feel stress in a set of situations. Please indicate how often you feel distressed or anxious in each of the following situations, on a scale of **1 (Never) to 5 (Very often)**. *

Please select the appropriate position for each element:

|  | **Never**  **(1)** | **Rarely**  **(2)** | **Occasionally (3)** | **Often (4)** | **Very often**  **(5)** | **Not applicable** | **I prefer not to answer** |
| --- | --- | --- | --- | --- | --- | --- | --- |
| **Situations related to problems with personal relationships** |  |  |  |  |  |  |  |
| **Situations related to family problems** |  |  |  |  |  |  |  |
| **Situations related to work-related problems** |  |  |  |  |  |  |  |
| **Situations related to financial problems** |  |  |  |  |  |  |  |
| **Situations related to academic problems** |  |  |  |  |  |  |  |
| **Situations related to domestic problems** |  |  |  |  |  |  |  |
| **Situations when I**  **am away from home** |  |  |  |  |  |  |  |
| **Events that do not go as planned** |  |  |  |  |  |  |  |

## [Q38]

The following questions are about **the month during the last 12 months** when you felt the worst emotionally.

During that month, how often have your emotions interfered, on a scale of **1 (Never) to 5 (Very often),** with... *

Please select the appropriate position for each element:

|  | **Never**  **(1)** | **Rarely**  **(2)** | **Occasionally (3)** | **Often (4)** | **Very often**  **(5)** | **Not applicable** | **I prefer not to answer** |
| --- | --- | --- | --- | --- | --- | --- | --- |
| **...your academic performance?** |  |  |  |  |  |  |  |
| **...your professional performance?** |  |  |  |  |  |  |  |
| **...your domestic chores?** |  |  |  |  |  |  |  |
| **…your social life?** |  |  |  |  |  |  |  |
| **...your relationship with friends and family?** |  |  |  |  |  |  |  |

[Q39]

The following questions will look at how you feel about different aspects of your life.

Please indicate for each of the following questions how often you feel this way, on a scale of **1 (Never) to 5 (Very often**).

How frequently... *

Please select the appropriate position for each element:

|  | **Never (1)** | **Rarely (2)** | **Occasionally (3)** | **Often (4)** | **Very often**  **(5)** | **Not applicable** | **I prefer not to answer** |
| --- | --- | --- | --- | --- | --- | --- | --- |
| **do you miss company?** |  |  |  |  |  |  |  |
| **do you feel left out?** |  |  |  |  |  |  |  |
| **do you feel isolated from others?** |  |  |  |  |  |  |  |

## [Q40]

The following questions will look at how you felt during the last 30 days.

Please indicate for each of the following questions how often you have felt this way in the last 30 days, on a scale of **1 (Never) to 5 (Always)**.

In the last 30 days, how often have you felt... *

Please select the appropriate position for each element:

|  | **Never (1)** | **Rarely**  **(2)** | **Occasionally (3)** | **Often**  **(4)** | **Very often (5)** | **Not applicable** | **I prefer not to answer** |
| --- | --- | --- | --- | --- | --- | --- | --- |
| **...exhausted for no reason?** |  |  |  |  |  |  |  |
| **...nervous?** |  |  |  |  |  |  |  |
| **...so nervous that nothing could calm you down?** |  |  |  |  |  |  |  |
| **...hopeless?** |  |  |  |  |  |  |  |
| **...restless or agitated?** |  |  |  |  |  |  |  |
| **...so restless that you couldn't stand still?** |  |  |  |  |  |  |  |
| **...depressed?** |  |  |  |  |  |  |  |
| **...in constant effort (you felt that everything required effort)?** |  |  |  |  |  |  |  |
| **...so sad that nothing could cheer you up?** |  |  |  |  |  |  |  |
| **...worthless?** |  |  |  |  |  |  |  |

# Perceptions of mental health

Many people have suffered (or are currently suffering) from a mental illness that affects their ability to be functional in a family, relational or professional context. Many different terms are used to refer to this condition: "mental illness", "mental health problem", "emotional disturbance", "psychological disturbance or disorder", "mental challenge", etc.

[Q41] Have you ever suffered from a mental health problem? *

 Select one of the following answers

Please select **only one** of the following options:

Yes, I am currently suffering or have suffered No, never

I prefer not to answer

### [Q41a]

This question looks at the stigma associated with mental health. Stigma can make people feel bad about something that is out of their control. Please note that the following statements **do not** necessarily represent the project researchers' view of mental health or mental illness.

In this question, the term "mental health problem" will be used. However, other terms such as mental health, mental disorder, mental health condition, psychological health, emotional well-being, among others, may be used.

Please indicate the extent to which you agree (or disagree with) each of the following statements, on a scale of **1 (Strongly disagree) to 5 (Strongly agree): ***: *

Only answer this question if the following conditions are true:

The answer is 'Yes, I currently suffer or have suffered' in question '57 [Q41]' (Have you ever suffered from a mental health problem?).

Please select the appropriate position for each element:

|  | **Strongly disagree**  **(1)** | **Disagree**  **(2)** | **I don’t know**  **(3)** | **Agree**  **(4)** | **Strongly agree**  **(5)** | **I prefer not to answer** |
| --- | --- | --- | --- | --- | --- | --- |
| **I think the stereotypes about people with mental health problems apply to me** |  |  |  |  |  |  |
| **I think that, in general, I can live life the way I want to** |  |  |  |  |  |  |
| **I think negative stereotypes about people with mental health problems make me feel isolated** |  |  |  |  |  |  |

|  | **Strongly disagree**  **(1)** | **Disagree**  **(2)** | **I don’t know**  **(3)** | **Agree**  **(4)** | **Strongly agree (5)** | **I prefer not to answer** |
| --- | --- | --- | --- | --- | --- | --- |
| **I think I feel out of place/ left out from the world because I have a mental health problem** |  |  |  |  |  |  |
| **I think being surrounded by people who do not have a mental health problem makes me feel out of place or maladjusted** |  |  |  |  |  |  |
| **I don't think people without a mental health problem can understand me.** |  |  |  |  |  |  |
| **I think that because I have a mental health problem, no one would be interested in coming close to me** |  |  |  |  |  |  |
| **I think that because I have a mental health problem, I cannot contribute to society** |  |  |  |  |  |  |
| **I think I can lead a full and fulfilling life despite my mental health problem** |  |  |  |  |  |  |

|  | **Strongly disagree**  **(1)** | **Disagree**  **(2)** | **I don’t know**  **(3)** | **Agree**  **(4)** | **Strongly agree (5)** | **I prefer not to answer** |
| --- | --- | --- | --- | --- | --- | --- |
| **I think that in my culture it is acceptable to receive treatment for mental health problems** |  |  |  |  |  |  |
| **I prefer not to answer** |  |  |  |  |  |  |

## [Q42]

The next question will look at your views and opinions of **people with a mental illness**. In this section, the term 'mental health problem' will be used. However, other terms such as mental illness, mental disorder, psychological disorder, or mental health condition may be used.

Please indicate the extent to which you agree (or disagree with) each of the following statements, on a scale of **1 (Strongly disagree) to 5 (Strongly agree): ***: *

Please select the appropriate position for each element:

|  | **Strongly disagree**  **(1)** | **Disagree (2)** | **I don’t know (3)** | **Agree (4)** | **Strongly agree (5)** | **I prefer not to answer** |
| --- | --- | --- | --- | --- | --- | --- |
| **Most people believe that people with a mental health problem could overcome the problem if they wanted to.** |  |  |  |  |  |  |
| **Most people believe that having a mental illness is a sign of personal weakness.** |  |  |  |  |  |  |
| **Most people believe that mental illness is not a real disease.** |  |  |  |  |  |  |
| **Most people believe that people with mental health problems are dangerous.** |  |  |  |  |  |  |

|  | **Strongly disagree**  **(1)** | **Disagree (2)** | **I don’t know (3)** | **Agree (4)** | **Strongly agree**  **(5)** | **I prefer not to answer** |
| --- | --- | --- | --- | --- | --- | --- |
| **Most people believe that it is better to avoid people with a mental illness so that they don't become mentally ill too.** |  |  |  |  |  |  |
| **Most people believe that people with mental health problems are unpredictable.** |  |  |  |  |  |  |
| **Most people would not tell anyone if they had a mental health problem.** |  |  |  |  |  |  |
| **Most people would not hire someone if they knew they had a mental illness.** |  |  |  |  |  |  |
| **I think I can lead a full and fulfilling life despite my mental health problem.** |  |  |  |  |  |  |
| **Most people would not vote for a politician if they knew they had a mental illness.** |  |  |  |  |  |  |

|  | **Strongly disagree**  **(1)** | **Disagree (2)** | **I don’t know (3)** | **Agree (4)** | **Strongly agree (5)** | **I prefer not to answer** |
| --- | --- | --- | --- | --- | --- | --- |
| **I prefer not to answer.** |  |  |  |  |  |  |

[Q43]

What else would you like to tell us? This includes, but is not limited to, for example, sharing more information about your mental health or wellbeing, coping strategies and resources or use of (and interest in) technology.

*If you prefer not to answer, you can leave this space blank.*

Please write your answer here:

Should you wish to be contacted to receive a summary of the results arising from this survey in a published study, please complete this form [(https://inqueritos.up.pt/index.php?r=survey/index&sid=489829&lang=pt)](https://inqueritos.up.pt/index.php?r=survey/index&sid=489829&lang=pt).

Thank you for your participation and contribution to the research!

Thank you for completing this survey.
