# Supplementary material for: Attitudes of Physicians and Individuals Toward Digital Mental Health Tools: Protocol for a Web-Based Survey Research Project
Source: JMIR Res Protoc. 2023 Mar 14;12:e41040. doi: 10.2196/41040 (PMC10131781; doi:10.2196/41040)
Supplement: Multimedia Appendix 3 [file resprot_v12i1e41040_app3.docx]

**Multimedia Appendix 3 – Checklist for a future researcher**

This Appendix was conceived to further the comparability of results obtained from carrying out this research protocol in different settings, as well as to enhance verification by reproducibility, which is indispensable to science.

As such, and aside from listing the methods that were adopted in the protocol’s text, the authors have compiled a set of tasks that had to be checked before the study could be considered fit for implementation and its results consistent.

It is our sincere hope that it may help you.

**Items to be checked:**

- Take the final survey questionnaires made available on Dahlhausen’s Multimedia Appendix 2 and in Borghouts Multimedia Appendix 1 as given.
- Translate these survey questionnaires to your native language using licensed translators.
- Gather feedback on translated questionnaires – considering a baseline of 10 individuals who may report important input for the research – to consider important questions in using digital health tools in your context as well as to the overall mental health care landscape and that of anxiety and depression.
- Incorporate obtained feedback by consensus and produce survey questionnaires of your own.
- Make sure these questionnaires adhere to CHERRIES if they are deployed online.
- Translate your own questionnaires to English using a certified translator to enlarge the knowledge corpus on the topic as well as promote reproducibility.
- List the potential number of people your survey will gather information from.
- List any institutional support you receive in conducing these surveys.
- Establish privacy mechanisms to enhance response to the survey, along with your inclusion and exclusion criteria.
- Specify in advance how gathered data will be analyzed both for variables you expect to stay the same and those you expect to change, and why.
